# Virtual commissioning of the Korea Light Source Hard X-ray Nanoprobe beamline with multilingual natural language control

**DOI:** 10.1107/S1600577526006788

**Published:** 2026-07-31

**Authors:** Minho Seo, Hyeong-joo Choi, Daseul Ham, Changwan Ha, Yong-Hyun Kim, Changhui Lee, Jun Lim, Ki-jeong Kim, In-hui Hwang, Jaeyong Shin

**Affiliations:** a 4GSR Research Division, Pohang Accelerator Laboratory, POSTECH, Pohang, Gyeongbuk37673, Republic of Korea; b Research Center for Beamline Korea Basic Science Institute Yuseong-gu Daejeon34133 Republic of Korea; c PLS-II Beamline Department, Pohang Accelerator Laboratory, POSTECH, Pohang, Gyeongbuk37673, Republic of Korea; d Division of Advanced Nuclear Engineering, POSTECH, Pohang, Gyeongbuk37673, Republic of Korea; ESRF – The European Synchrotron, France

**Keywords:** virtual commissioning, beamline simulation, Monte Carlo ray tracing, *EPICS*, *Bluesky*, natural language processing, hard X-ray nanoprobe

## Abstract

Commissioning of a synchrotron hard X-ray nanoprobe beamline traditionally requires months of iterative alignment after hardware installation, during which operational knowledge accumulates but remains inaccessible to non-specialist users. To address this, we present a browser-based virtual commissioning platform for the Korea Light Source ID10 Hard X-ray Nanoprobe beamline (first light 2029) that allows beamline scientists to design, test and refine alignment procedures, scan plans and experimental workflows years before the first photon arrives. Specifically, the platform integrates a Monte Carlo ray-tracing engine, a standard *Experimental Physics and Industrial Control System* (*EPICS*)/*Bluesky* control stack, and a multilingual natural-language interface within a single deployable package, which we name HANBIT (Hybrid Agent-driven Natural-language Beamline Interactive Toolkit). Users can interactively explore parameter trade-offs, such as the effect of the secondary source aperture on beam size versus photon flux. The Monte Carlo engine reproduces the overall *Shadow4* beam-profile shape and is validated against *SPECTRA* undulator spectra, source size and divergence. The natural language processing (NLP) agent achieves 98.2% automated action-identification accuracy across 228 test cases in Korean, English and Japanese, whereas expert review of the same responses yields an acceptance rate of 67.3%. We identify this 30.9 percentage-point gap as a central finding: automated accuracy does not guarantee operational acceptability, and closing it is the key challenge for deployment-grade natural-language beamline control. We further validate the zero-change hardware transition strategy that the beamline pursues on three real hardware subsystems, confirming that at the validated device layers the control code and scan plans operate unchanged on the real devices; the integration of the remaining parts, such as high-rate area detectors, which awaits the detector hardware, is also discussed.

## Abstract 2

A pre-installation virtual commissioning platform for the Korea Light Source ID10 hard X-ray nanoprobe couples browser-based Monte Carlo ray tracing, *EPICS*/*Bluesky* control and a multilingual natural-language agent (98.2% accuracy in Korean, English and Japanese) under a zero-change hardware transition principle, producing commissioning design inputs ahead of 2029 first light.

## 1. Introduction

**Figure 1:**
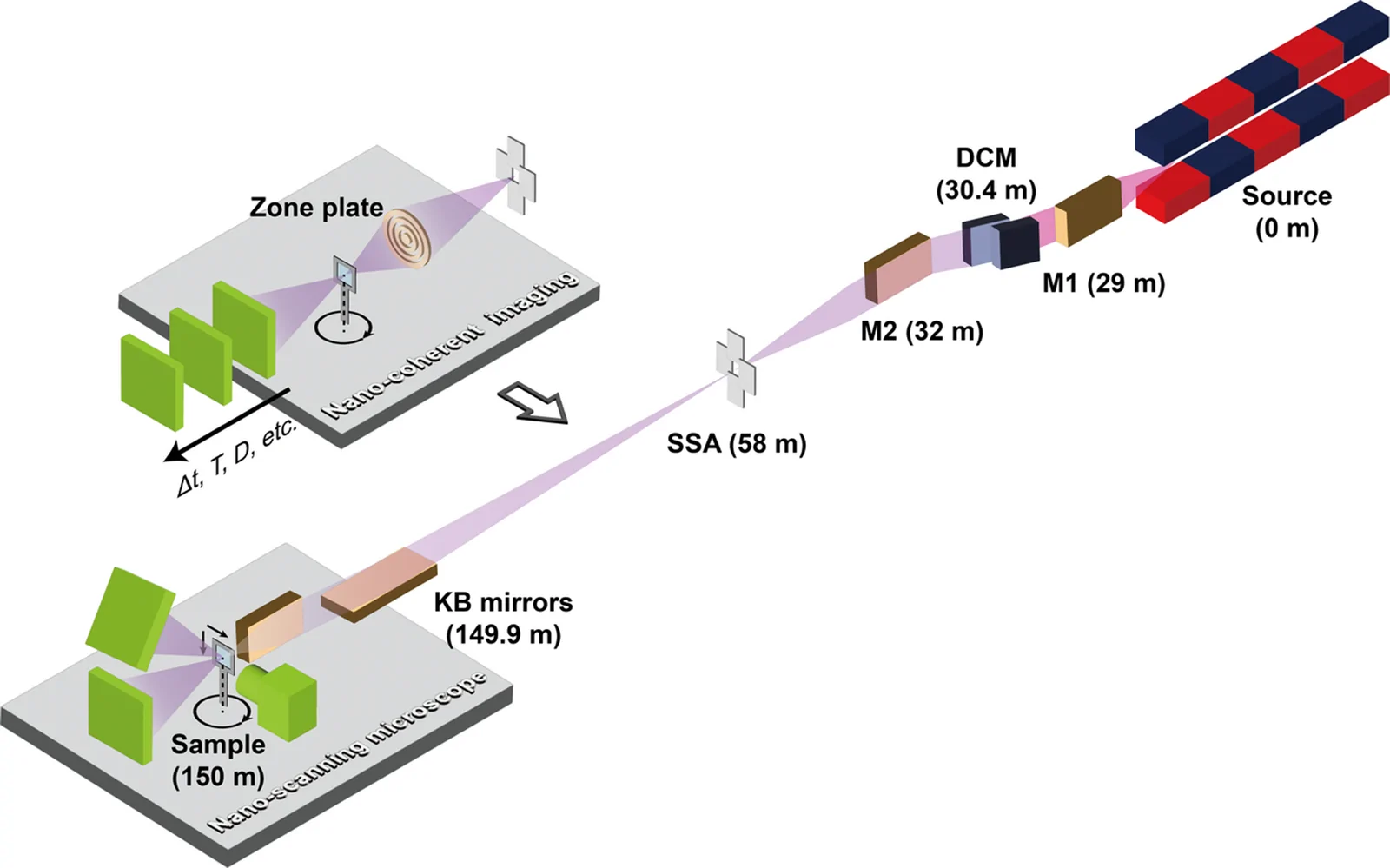
Schematic optical layout of the KLS ID10 Hard X-ray Nanoprobe beamline (source to sample, 150 m). The IVU24 in-vacuum undulator produces X-rays at 5–25 keV. Two grazing-incidence mirrors (M1, M2) provide harmonic rejection and vertical/horizontal focusing. A Si(111) double-crystal monochromator (DCM) selects the photon energy with Δ*E*/*E* ≃ 1.3 × 10^−4^. The secondary source aperture (SSA) at 58 m defines the virtual source for Kirkpatrick–Baez (KB) nano-focusing mirrors, which deliver a ∼50 nm focused beam at the sample position. Source parameters and component positions are listed in Tables S7 and S8, respectively.

Fourth-generation synchrotron light sources based on multi-bend achromat (MBA) lattices deliver X-ray beams of unprecedented brilliance, enabling nanometre-scale probes at hard X-ray energies (Eriksson *et al.*, 2014 ▸; Raimondi, 2016 ▸; Borland *et al.*, 2014 ▸). The Korea Light Source (KLS; formerly Korea-4GSR), a 4.0 GeV MBA storage ring with a horizontal emittance of 62 pm rad, is currently under construction with the first light anticipated in 2029 (Korea-4GSR Project Team, 2024 ▸). Among the initial beamlines, ID10 (*EPICS* control-system prefix BL10) is a Hard X-ray Nanoprobe (HXNP; Fig. 1 ▸) designed to deliver a focused beam of ∼50 nm spot size across the 5–25 keV energy range. The beamline comprises an IVU24 in-vacuum undulator (Ha *et al.*, 2024 ▸) as the photon source, two grazing-incidence mirrors (M1 and M2) for harmonic rejection and horizontal and vertical focusing, a double-crystal monochromator (DCM), a secondary source aperture (SSA), and Kirkpatrick–Baez (KB; Kirkpatrick & Baez, 1948 ▸) nano-focusing mirrors. Commissioning such a complex optical chain is time-consuming and traditionally requires iterative physical alignment cycles once the hardware is installed.

The KLS HXNP beamline faces three commissioning challenges. First, the project allocates approximately six months for first-light commissioning in 2029; any procedure validated before real photons arrive directly compresses this schedule. Our platform addresses this through a three-phase approach: virtual commissioning with a complete software replica of the beamline (Phase 1, 2026–2027), progressive integration of the beamline hardware as the optical components, detectors, data server, and control systems are installed (Phase 2, 2027–2029), and production operation from first light (Phase 3, 2029). Second, the required software tools—optics simulation codes [ray tracing: *Shadow4* (Sanchez del Rio *et al.*, 2025 ▸); wavefront propagation: *SRW* (Chubar & Elleaume, 1998 ▸)], control systems (*EPICS*), and data-acquisition frameworks (*Bluesky*)—are developed independently and do not interoperate within a single environment, making virtual commissioning impractical with existing tools. Third, synchrotron users are typically sample scientists, not X-ray experts; at an international facility, language compounds this expertise barrier.

To overcome these challenges, we intend to leverage digital twins and natural language processing (NLP) agents. We use the term ‘digital twin’ in the broader sense of Grieves & Vickers (2017 ▸): a virtual representation that serves as the real-time digital counterpart of a physical system. More precisely, our system is a pre-installation virtual commissioning platform that replicates the full control stack [*EPICS* (*Experimental Physics and Industrial Control System*) process variables, PVs; *Ophyd* devices; *Bluesky* scan plans] before the physical beamline exists, enabling software development and procedure validation years ahead of hardware installation. We emphasize that this is a virtual commissioning framework rather than a high-fidelity industrial digital twin: it does not yet provide bidirectional state synchronization with operating hardware, predictive-maintenance modeling, or full-fidelity physical representation, and we reserve those capabilities for future work once the physical beamline exists. The concept of a digital twin has been applied to beamline instrumentation by several groups. The BESSY II initiative integrates the *RAY-UI* ray tracer with *Bluesky* via the *RayPyNG-Bluesky* package (Vadilonga *et al.*, 2023 ▸; Vadilonga *et al.*, 2025 ▸), but requires a server-side simulation engine and does not embed optics computation in the browser client. The ESRF *Daiquiri* framework (Fisher *et al.*, 2021 ▸) similarly provides a web-based beamline-control user interface but does not embed an optics-physics model. Desktop simulation suites such as the *SHADOW* family (*SHADOW3*, *ShadowOui* and *SHADOW4*; Sanchez del Rio *et al.*, 2011 ▸; Rebuffi & Sanchez del Rio, 2016 ▸; Sanchez del Rio *et al.*, 2025 ▸) and *OASYS* (Rebuffi & Sanchez del Rio, 2017 ▸) provide accurate ray tracing but are disconnected from the control layer. On the control-system side, *Bluesky* and *EPICS* (Allan *et al.*, 2019*a* ▸; Dalesio *et al.*, 1994 ▸) offer robust data acquisition and device abstraction but carry no physics model of the beamline optics. The HEPS *Mamba* system (Liu *et al.*, 2022 ▸) provides an integrated beamline software solution but focuses on experiment control and data acquisition rather than physics simulation. Few systems bridge simulation, control, and user interaction within a single framework. Meanwhile, large language model (LLM) agents are emerging as natural-language interfaces for scientific instruments. The VISION system at NSLS-II demonstrated natural-language instrument control through a modular AI assistant (Mathur *et al.*, 2025 ▸), and HEPS has reported an LLM-based assistant integrated with *Mamba* (Bi *et al.*, 2024 ▸). More broadly, autonomous data acquisition using Gaussian processes (Noack *et al.*, 2021 ▸), AI-driven crystallography agents (Maffettone *et al.*, 2021 ▸) and facility-wide machine-learning roadmaps (Campbell *et al.*, 2021 ▸) demonstrate the growing role of AI in synchrotron science. However, none of these systems combine physics simulation, real-time *EPICS* control, and multilingual natural language operation within a single integrated framework (Table 1 ▸).

In this paper, we present HANBIT, a virtual commissioning platform that combines:

(i) A browser-based Monte Carlo ray-tracing engine for real-time optics simulation.

(ii) A standard *EPICS* Channel Access interface via a *caproto* soft input/output controller (IOC).

(iii) The *Bluesky* data-acquisition framework with *Ophyd* devices.

(iv) A multi-backend multilingual NLP agent for non-expert beamline operation.

The architecture is designed so that the same front-end code, *Ophyd* device definitions and scan plans operate unchanged whether driven by the virtual soft IOC or a real hardware IOC. We call this the zero-change hardware transition strategy, a deliberate construction choice for this beamline. To verify its feasibility, we demonstrate it at the motor-record and scalar-readout device layers across three real hardware subsystems. Extending it to the remaining layers, such as high-rate area-detector integration, which additionally requires the detector hardware and a dedicated high-throughput data server, is Phase 2 work. The system is implemented as a JavaScript front-end and a Python server back-end. The remainder of this paper is organized as follows. Section 2 presents the system architecture and control integration. Section 3 details the Monte Carlo physics engine. Section 4 presents the multilingual NLP agent. Section 5 describes virtual commissioning procedures and demonstrates full-stack operation. Section 6 summarizes and discusses the outlook.

## 2. System architecture and control integration

**Figure 2:**
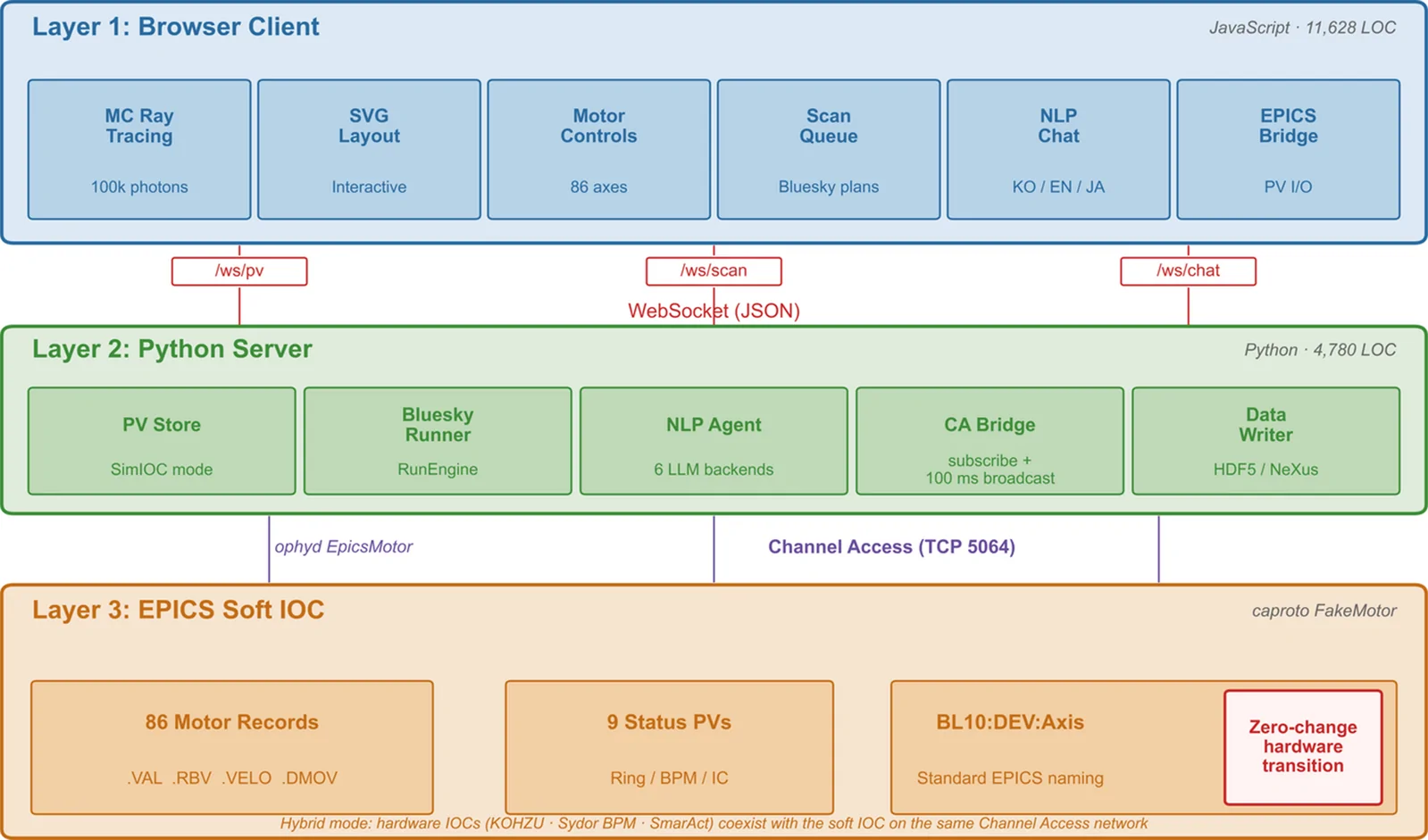
Three-layer system architecture. Layer 1: the browser client renders the interactive beamline layout, Monte Carlo beam profile, motor controls, scan queue, NLP chat interface, and an *EPICS* bridge using vanilla ES5 JavaScript with zero external dependencies. Layer 2: a Python WebSocket server provides three endpoints (/ws/pv for *EPICS* PV read/write/subscribe, /ws/scan for *Bluesky* plan submission and event streaming, /ws/chat for NLP agent communication). Layer 3: a *caproto* soft IOC serves 86 *EPICS* motor-record PVs and 9 status PVs via Channel Access.

The virtual commissioning platform follows a three-layer architecture (Fig. 2 ▸):

Layer 1—browser client: domain-organized JavaScript modules implementing the physics engine, interactive SVG (Scalable Vector Graphics; W3C, 2011 ▸) beamline layout, motor controls, scan queue, and NLP chat interface. We use vanilla ES5 (ECMAScript 5; ECMA International, 2009 ▸) without external framework dependencies, ensuring zero-installation deployment on any browser. This choice prioritizes deployment simplicity in beamline network environments where external package sources and build toolchains may be restricted, and it provides a natural host for the NLP chat interface (Section 4).

Layer 2—Python server: WebSocket hub with three endpoints: /ws/pv for *EPICS* process-variable (PV) read/write/subscribe, /ws/chat for NLP agent communication, and /ws/scan for *Bluesky* scan submission and event streaming.

Layer 3—*EPICS* infrastructure: a soft input/output controller (IOC) implemented with *caproto* (Allan *et al.*, 2019*b* ▸), a pure-Python implementation of the *EPICS* Channel Access protocol requiring no compilation, serving 86 standard motor records and 9 status PVs via Channel Access on port 5064. In the current hybrid configuration the soft IOC serves 83 of these axes; the three sample-stage axes are provided by the KOHZU hardware IOC instead.

From a user’s perspective, the virtual beamline behaves identically to a real *EPICS* installation: every motor responds to the standard motor–record interface, status channels simulate realistic noise [ring current jitter, beam position monitor (BPM) position noise, ion-chamber dark current], and the complete *Bluesky* data-acquisition stack (Allan *et al.*, 2019*a* ▸) runs unmodified through *Ophyd* EpicsMotor devices (Arkilic *et al.*, 2017 ▸). The *caproto* soft IOC (Allan *et al.*, 2019*b* ▸) serves 86 motor-record PVs across 14 device groups, and the Python server bridges the IOC to the browser client via an event-driven Channel Access bridge built on *caproto* subscribe callbacks, broadcasting PV changes to the browser on a periodic loop. The median latency is approximately 30 ms on localhost, a software floor; the realistic remote-network path is about 91 ms (see Section S1 of the supporting information). This latency is bounded by the server-side 10 Hz broadcast loop rather than by Channel Access; the bridge is not on a latency-critical control path (feedback loops remain in *EPICS* and hardware).

We achieve this zero-change transition by:

(i) Using *caproto* FakeMotor with record = ‘motor’, which provides the full *EPICS* motor-record field set (.VAL, .RBV, .VELO, .DMOV, .HLM, .LLM, .EGU, *etc*.).

(ii) Using *Ophyd* EpicsMotor directly (no custom wrapper).

(iii) Maintaining a consistent PV naming convention (*e.g.* BL10:SAM:CX, BL10:DCM:Theta) between soft and real IOCs.

The practical consequence of this design is that three years of software development, scan-plan debugging, and operator training can proceed in parallel with hardware procurement, and the procedures validated on the virtual platform are designed to transfer to the real beamline without code changes at the motor-record and scalar-readout device layers. Concretely, these layers are the browser client, the *Ophyd* EpicsMotor and scalar-readout device definitions, and the *Bluesky* scan-plan logic, which we demonstrate below to transfer to real hardware without modification. The other layers (the full optics-chain motion hardware, high-rate area detectors, timing-sensitive feedback loops, detector streaming under realistic load, and failure recovery) are not yet validated and are addressed as Phase 2 work, which we will bring up in simulation first by extending the soft-IOC stand-in approach already used here for the motor and scalar devices and by adopting the standard simulated IOCs that already exist for these layers, so that their *Ophyd* device definitions and *Bluesky* scan plans can be exercised and hardened before the corresponding hardware comes online. We validated the zero-change strategy with three real hardware subsystems on a test network: a KOHZU three-axis sample stage (ARIES controller) via *EPICS* motor IOC, a Sydor diamond beam position monitor via synApps quadEM IOC, and a SmarAct MCS2 nano-positioner with PicoScale laser interferometer via TCP bridge (Fig. S1 of the supporting information). In all three cases, the browser client, *Ophyd* devices, and *Bluesky* scan plans operated without code modification; the only configuration change was the *EPICS* Channel Access address list for the two *EPICS*-served subsystems and the TCP bridge endpoint for the nano-scanner. Detailed specifications, IOC configurations, and end-to-end test results are provided in Section S2 of the supporting information. The system supports a hybrid mode in which these hardware IOCs (KOHZU, Sydor BPM, SmarAct) coexist with the *caproto* soft IOC on the same Channel Access network, allowing already-validated subsystems to run on real hardware while the remaining subsystems remain in simulation; the operating mode is depicted in Layer 3 of Fig. 2 ▸.

The present validation focuses on motors and scalar diagnostics; a production HXNP beamline will additionally operate one or more high-speed area detectors (*e.g.* EIGER2 X or PILATUS). Integrating such detectors will require additional code on both the *EPICS* side [an *areaDetector* (Rivers, 2010 ▸) IOC for control-rate integration, or a higher-throughput framework such as *Odin* (Yendell *et al.*, 2019 ▸) for the fastest detectors] and the *Bluesky*/*Ophyd* client side, and raises data-rate and data-storage considerations that the present motor and scalar layer does not exercise. Such detectors can generate raw data streams of order 1–10 GB s^−1^, well beyond what the present Channel Access bridge and file-based event output were designed to carry, so a production deployment will require a dedicated detector data path (for example an *areaDetector* pipeline with a dedicated file-writer plugin, or an *Odin* pipeline with its parallel file writers) separate from the control path demonstrated here. During this Phase 2 detector integration an *EPICSareaDetector* ADSim IOC will stand in for the future detector IOCs, letting us develop and test this dedicated data path, including its high-rate data handling and streaming, under realistic gigabyte-per-second loads before the hardware exists.

All client–server communication uses the WebSocket protocol (RFC 6455; Fette & Melnikov, 2011 ▸) with JSON (JavaScript Object Notation)-encoded messages.

**PV endpoint (/ws/pv).** Supports subscribe, get, and put actions. The server broadcasts changed PV values to subscribed clients at 100 ms intervals; the token-based authentication required for production use is discussed in Section 4.

**Scan endpoint (/ws/scan).** Actions: submit (run a single *Bluesky* plan), abort, pause, resume, status, list plans. The server broadcasts *Bluesky* document stream events (start, descriptor, event, stop) to all connected scan clients in real time. Plans execute one at a time on a single in-process RunEngine (a concurrent submission is rejected rather than queued); the scan queue is a client-side construct in the browser and NLP layer, which keeps the server a single dependency-free deployable. This in-process bridge is not a managed multi-user queue; adopting *bluesky-queueserver* (a separate-process RunEngine Manager with an allowed-plans registry) is the planned path for the multi-user operational phase.

**Chat endpoint (/ws/chat).** Forwards user natural-language messages to the NLP agent and streams responses back to the client.

The *Bluesky* scan layer exposes five scan plans via the /ws/scan endpoint:

(i) **Energy scan**—linear scan of DCM θ with automatic energy-to-angle conversion via Bragg’s law (λ = 2*d*sinθ), where the wavelength λ and photon energy *E* are related by λ = *hc*/*E* (*hc* = 12.398 keV Å).

(ii) **X-ray absorption fine-structure (XAFS) scan**—multi-region step scan (step sizes: pre-edge 5 eV; edge 0.2–0.5 eV depending on the DCM angular resolution at the target energy, post-edge 2 eV) for elements from Ti to Pb across *K* and *L* edges.

(iii) **Raster scan**—two-dimensional grid scan of sample piezo stages (SX/SY) with optional virtual X-ray fluorescence (XRF) detector readout.

(iv) **Alignment scan**—one-dimensional peak scan on any motor axis with Gaussian/Lorentzian fitting.

(v) **Beam check**—multi-point intensity monitoring using ion chambers and BPMs.

Scan events are broadcast in real time to all connected WebSocket clients, enabling live chart updates in the browser. Data are streamed incrementally to NeXus (Könnecke *et al.*, 2015 ▸) / Hierarchical Data Format 5 (HDF5) files during acquisition, following the /entry/{instrument, sample, data} hierarchy for compatibility with the KLS facility data infrastructure. An SQLite database records scan metadata persistently, enabling history retrieval across server restarts. The browser client provides CSV, JSON, and PNG export for immediate data inspection. For the current single-session development phase, which has no multi-user data-isolation requirement and no authentication layer yet in place (Section 4), we write scan output directly to these standard NeXus/HDF5 files with an SQLite metadata index rather than running a separate data-access server. The *Bluesky*-ecosystem Tiled client-server application (Rakitin *et al.*, 2022 ▸) provides the API-based access (metadata search, partial reads and access-controlled multi-user retrieval) that a production facility needs; because it exposes a network data service, its responsible deployment is tied to the authentication and authorization layer, which is pending the facility’s operational access-control policy (Section 4). Tiled can serve the standard NeXus/HDF5 files that the platform already produces, so adopting it in production adds a server layer rather than requiring data reformatting, and we plan this migration for operational deployment.

## 3. Monte Carlo physics engine

### 3.1. Ray-tracing architecture and interactive use

The physics engine traces 50000 to 500000 photons per simulation cycle through the complete optical chain. Each ray is represented as a vector *r* = (*x*, *y*, *vx*, *vy*, *vz*, *w*), where (*x*,*y*) is the transverse position, (*vx*,*vy*,*vz*) is the unit direction vector, and *w* is a weight factor. The engine is designed for real-time interactive feedback rather than as a replacement for dedicated simulation codes such as *Shadow4* (Sanchez del Rio *et al.*, 2025 ▸) or *SRW* (Chubar & Elleaume, 1998 ▸); it does not model thermal deformation, mirror slope errors, or coherent wavefront propagation. The value of our engine lies in sub-second to few-second turnaround enabling interactive parameter exploration that is impractical with conventional codes.

Fig. 1 ▸ shows the optical layout. The beamline extends 150 m from source to sample (storage-ring and undulator source parameters are provided in Table S7 of the supporting information; optical element positions in Table S8 of the supporting information). Rays propagate sequentially through: source → masks → white-beam (WB) slit → M1 → DCM → M2 → SSA → KB-V → KB-H → sample. At each element, the ray position is updated by free-space drift, the direction is modified by reflection or diffraction, and the weight is reduced by the element’s transmission function.

The sub-second turnaround of the Monte Carlo engine transforms it from a passive validation tool into an interactive design aid. For example, a sample scientist can vary the SSA aperture from 10 to 200 µm and observe in real time how the focused spot size and photon flux at the sample respond, mapping the trade-off between spatial resolution and count rate before the SSA hardware is procured. Similarly, switching the M2 mirror coating between Rh, Pt and Si stripes at a given energy reveals the harmonic rejection ratio and reflectivity penalty within seconds, directly informing the stripe-selection protocol for the installed mirror. During the KB alignment rehearsal described in Section 5.1, the pitch-scan range and step count were determined from virtual scans showing that a ±0.5 mrad window with 21 steps resolves the focus minimum with *R*^2^ > 0.99; these parameters will be adopted as the starting recipe for real-beam commissioning. In each case the simulation result provides quantitative feedback that can be incorporated into the beamline design documentation and operational procedures before any optical component is physically installed.

### 3.2. Physics models and validation

**Figure 3:**
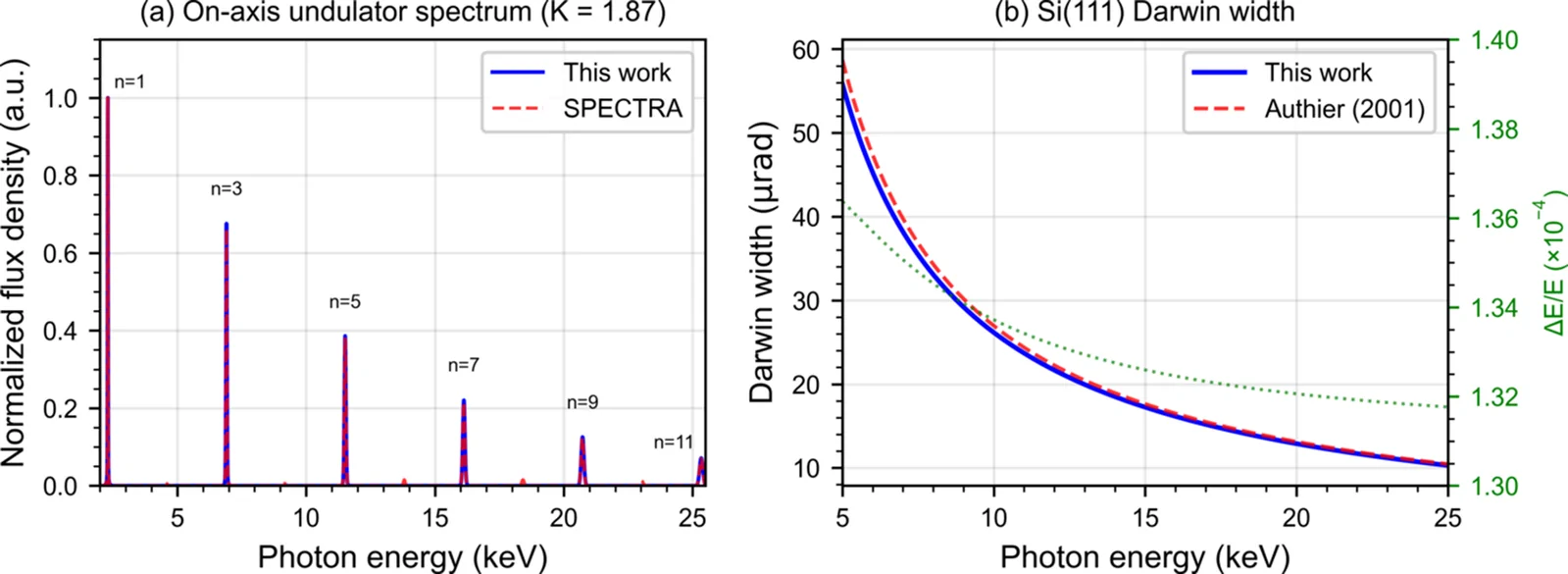
Physics engine validation against established codes. (*a*) On-axis undulator flux-density spectrum, normalized to the *n* = 1 peak and displayed from 2 keV so that harmonics *n* = 1, 3, 5, 7, 9 and 11 are all visible, at the representative operating point *K* = 1.87 (gap = 7.14 mm). This work (blue solid) reproduces the *SPECTRA *calculation (red dashed) for the peak flux density of every displayed harmonic, with the quantitative comparison given in Table 2 ▸. (*b*) Si(111) Darwin width versus photon energy: the engine (blue solid) reproduces the Authier (2001 ▸) analytical width [red dashed; Section S3.8, equation (S9)] over 5–25 keV (Table S10); the green dotted curve (right axis) gives the equivalent relative energy bandwidth Δ*E*/*E* (1.32–1.36 × 10^−4^).

**Figure 4:**
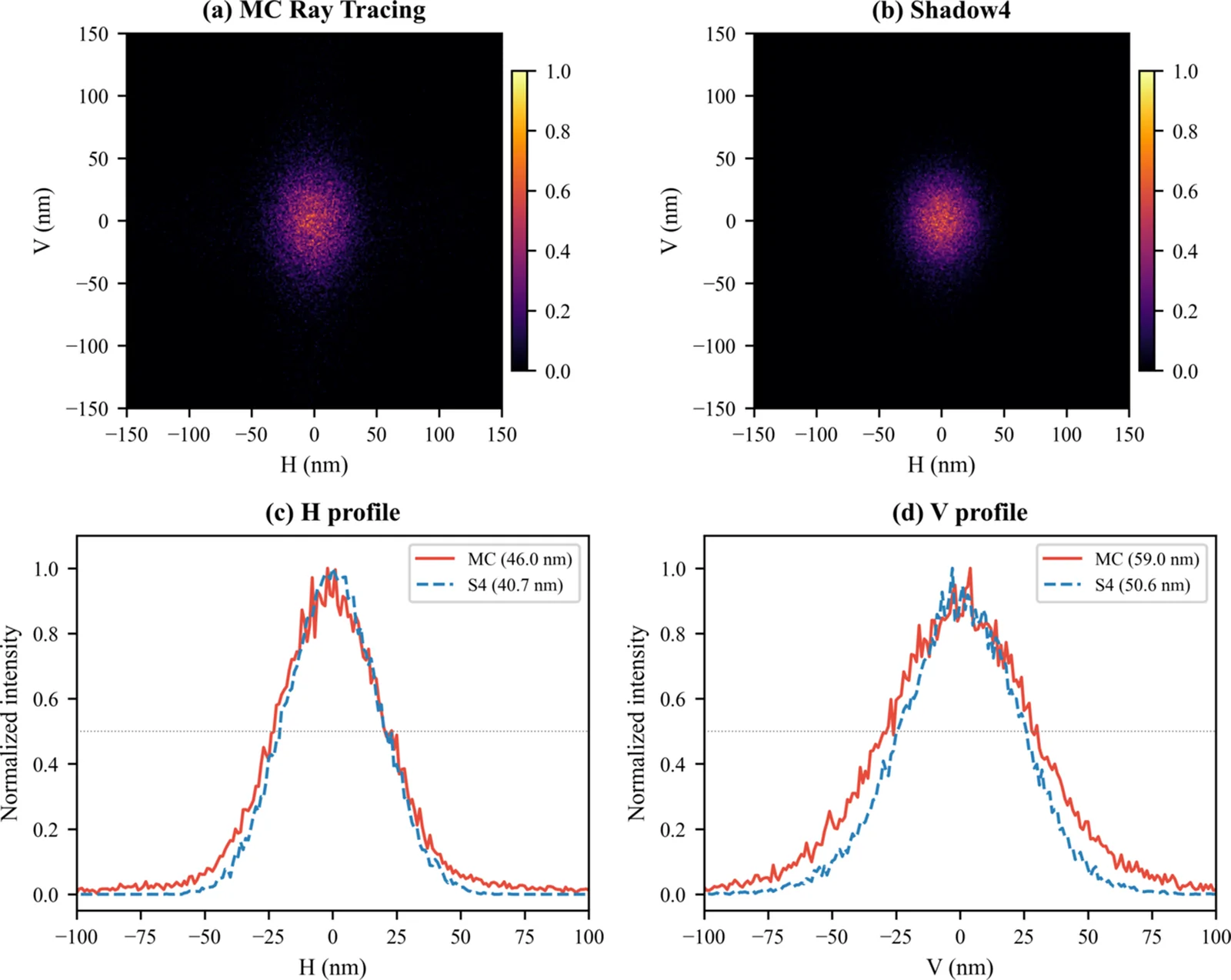
Monte Carlo (MC) beam profile at the sample position compared with *Shadow4* (*S4*) hybrid ray tracing (10 keV, SSA 50 µm × 50 µm, 500000 rays). (*a*) 2D intensity distribution from the MC engine. (*b*) 2D intensity distribution from *S4*. (*c*) Horizontal one-dimensional projected profile (integrated along the orthogonal axis): MC (red solid) versus *S4* (blue dashed), with FWHM values annotated. (*d*) Vertical one-dimensional projected profile (integrated along the orthogonal axis). The MC engine reproduces the overall beam shape with a systematic broadening relative to *S4*.

The on-axis photon flux from the IVU24 undulator is computed using the Kim formalism (Kim, 1989 ▸), with the angle-dependent harmonic amplitude re-implemented in JavaScript following the *SPECTRA* far-field formulation (Tanaka & Kitamura, 2001 ▸) and convolved over the electron-beam energy spread and divergence (Tanaka, 2014 ▸); the result is validated against the *SPECTRA* solver, Version 11 (Tanaka, 2021 ▸), for harmonics *n* = 1–15 over *K* = 1.0–2.5, including the source size and divergence (see Section S3 of the supporting information; Fig. S3 and Tables S11 and S12). Fig. 3 ▸(*a*) and Table 2 ▸ summarize the on-axis flux density of harmonics *n* = 3–11 and the flux through the white-beam slit, whose default opening of 1.2 mm × 1.2 mm at 27.8 m (±21.58 µrad) is the acceptance used in Table 2 ▸. Grazing-incidence mirror reflection uses Fresnel reflectivity with optical constants from DABAX tabulations based on Henke atomic scattering factors (Henke *et al.*, 1993 ▸), consistent with *xraylib* (Schoonjans *et al.*, 2011 ▸). The DCM model implements two sequential Bragg reflections using the Darwin reflectivity width (Zachariasen, 1945 ▸; Authier, 2001 ▸). The KB mirrors are modeled as ideal elliptical reflectors. The SSA produces Fraunhofer diffraction computed by coherent wavefront propagation using the discrete Fourier transform. The focused beam profile is the convolution of the demagnified source, SSA diffraction, and KB diffraction contributions (Section S3.10). Full physics model details are provided in Section S3. Statistical convergence was verified across 10000 to 500000 ray runs (Table S9): the operational default of 100000 rays completes in 200–500 ms and reproduces the 500000-ray reference full width at half-maximum (FWHM), while a 500000-ray reference run completes in 1–2 s. The Darwin width reproduces the Authier (2001 ▸) analytical reference, with crystal parameters from the crystalpy library (Guigay & Sanchez del Rio, 2024 ▸); a comparison over 5–25 keV is given in Fig. 3 ▸(*b*) and Table S10 [equation (S9) in Section S3.8]. Fig. 4 ▸ compares the Monte Carlo beam profile with *Shadow4* hybrid ray tracing (Shi *et al.*, 2014 ▸) at 10 keV.

The harmonic partial-flux calculation is GPU-accelerated through a four-layer cascade (a closed-form fast path for instantaneous display, WebGPU, a precomputed lookup table, and a CPU Web Worker); the three converged paths return the same *SPECTRA*-validated value with or without a GPU, as detailed in Sections S3.6 and S3.7 (fast-path-to-acceptance ratio in Table S13).

### 3.3. Accuracy scope and planned improvements

As noted above, the Monte Carlo physics engine is designed as an interactive commissioning approximation rather than a metrology-grade predictive tool, and its accuracy is correspondingly limited; the planned accuracy improvements are described at the end of this section. In its current version the physics engine does not answer to absolute accuracy the two quantitative commissioning questions of whether the beam focus is as small as expected and whether the absolute flux delivered to the sample is as high as expected. For focus size, coherent Fraunhofer diffraction is applied at the secondary source aperture and a hybrid Fresnel correction at the KB nanofocus. These are localized diffraction corrections only and do not perform the coherent wavefront propagation across the optical system required for rigorous nanofocus prediction. For absolute flux, the displayed detector signal remains a relative quantity derived from the on-axis density, not a calibrated absolute count rate; the detector-response chain required to convert it to an absolute count rate is outlined in Section S3.9, and introducing an established ionization-chamber response model, such as the flux calculator of *XAFSmass* (Klementiev & Chernikov, 2016 ▸), calibrated against measured ion-chamber currents, is identified as future work. In addition, both quantities are modeled for the nominal design source and an ideal undulator field, whereas the real electron-beam source size, divergence, undulator phase errors, and mirror shape errors are as-built properties not known at the design stage, so the absolute flux and focus-size values the engine reports are approximate predictions, to be reconciled with the machine during real commissioning. To prevent over-interpretation by non-specialist users, the application also displays this accuracy-scope notice as a startup dialog in the graphical user interface, stating that the beam-size and flux values are approximate and intended for interactive exploration rather than absolute prediction.

The Monte Carlo ray tracing currently runs CPU-only in single-threaded JavaScript (the harmonic flux calculation is GPU-accelerated, as described above), which bounds the achievable ray count and optical complexity. Within these limits the physics engine is well suited to standard commissioning tasks such as rehearsing alignment procedures, validating scan plans, exploring parameter trends such as the trade-off between secondary-source aperture and beam size or flux, and training operators; tasks that demand absolute focus-size or flux prediction, coherence propagation, and slope-error or wavefront studies continue to require conventional optics codes.

Building on this, two further improvements are planned: extending to the Monte Carlo ray count itself the same GPU acceleration (WebGPU or WebAssembly) already applied to the flux calculation, together with an optional high-fidelity backend that exports the configured optical layout to *SRW* for wavefront propagation and to *xrt* (Klementiev & Chernikov, 2014 ▸) for ray tracing; and, once the beamline is operational, calibration of the modeled beam quantities such as focus size and flux against values measured during real commissioning, so that the engine’s accuracy improves with operational data rather than remaining fixed at installation. Because the browser is deliberately limited to this interactive-approximation layer, with computations beyond interactive client capacity (greater optical complexity, or absolute prediction) offloaded to that backend rather than scaled in the client, and because the server is a single dependency-free deployable that also supports hybrid real-plus-simulated operation (Section 2), the platform is designed to remain operationally sustainable as optical complexity grows.

## 4. Multilingual NLP agent

### 4.1. Action pipeline and prompt design

To address the experiment accessibility gap identified in Section 1, we developed a natural language interface that translates plain-language requests—in the user’s native language—into validated beamline actions. The NLP agent supports six LLM backends: Ollama (local Qwen3-32B, free/offline), Google Gemini 2.0 Flash (free tier), Groq Llama 3.3 70B (free, low latency), Anthropic Claude Sonnet 4.6, vLLM with Qwen3-32B and prefix caching (recommended production backend), and Solar Pro3 (Korean-optimized, low latency). This multi-backend approach provides resilience against API outages and allows the facility to select the backend that best balances cost, latency, and quality.

**Figure 5:**
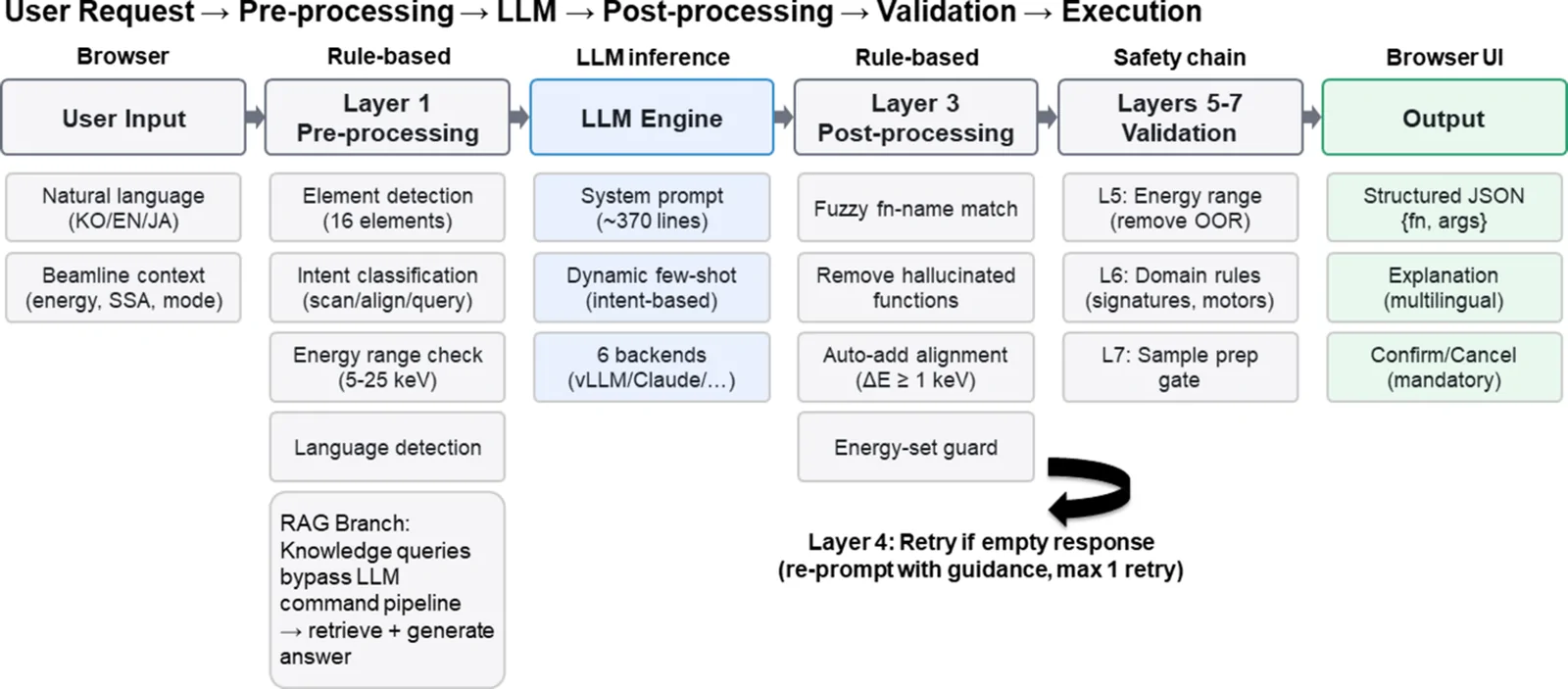
Seven-layer NLP action pipeline. User input in natural language (Korean, English, or Japanese) is pre-processed in Layer 1 (element/edge detection, intent classification, language auto-detection, knowledge-query routing) and sent to the selected LLM backend (Layer 2). The raw output then passes through five post-processing layers (Layers 3–7) that perform fuzzy function-name matching against 47 registered beamline functions, empty-response retry, energy-range validation, domain rule enforcement (function-signature checks, motor-group constraints, scan-motor compatibility), and a sample-preparation gate that blocks measurement actions until the user confirms that a physical sample is mounted and aligned. Knowledge-query inputs are routed instead to a retrieval-augmented generation branch, bypassing the command pipeline. The entire pipeline is deterministic and stateless so that each layer can be debugged and extended independently.

The NLP agent processes user requests through a seven-layer action pipeline (Fig. 5 ▸). Layer 1 (pre-processing) analyzes the input text before the LLM call: it detects element symbols and absorption-edge energies for 16 elements (Ti through Pb), classifies the user intent into one of 12 categories (*e.g.* scan, alignment, motor control, knowledge query), checks whether referenced energies fall within the beamline’s 5–25 keV operating range, and auto-detects the input language via Unicode script analysis (Hangul, Katakana/Hiragana, Chinese, Arabic, Thai, Devanagari) with Latin-script keyword fallback. Knowledge queries (*e.g.* ‘what is the beam size at 15 keV?’) are routed to a retrieval-augmented generation (RAG) branch that bypasses the command pipeline entirely: the query is embedded and matched against a pre-indexed vector store of beamline documentation (specifications, motor ranges, technique descriptions), and the top-five retrieved chunks are injected into the LLM context to generate a factual, source-attributed answer.

The system prompt (∼370 lines for models ≥ 32 B parameters, ∼180 lines for smaller models) embeds beamline-specific domain knowledge including absorption-edge energies, experimental technique templates, motor-group aliases, and trilingual vocabulary. Before each LLM call, a dynamic prompt composer selects relevant few-shot examples based on the detected intent—for instance, an alignment intent triggers alignment-specific examples while suppressing scan examples—reducing prompt length and improving action accuracy for the target task. Layer 2 (LLM inference): the composed prompt—system instructions, dynamic few-shot examples, beamline context, and Layer 1 hints—is sent to the selected backend, which generates a structured JSON response containing action function names, arguments, and a natural-language explanation.

The raw LLM output passes through five post-processing layers (Layers 3–7) before reaching the user. Layer 3 (post-processing) performs fuzzy matching of function names against the 47 registered beamline functions spanning motor control, scan execution, alignment procedures, energy/optics setting, and hardware status queries, removes hallucinated function calls, and applies context-aware corrections: if the user requested only an energy change (not a scan), any scan actions are replaced with the appropriate setTargetEnergy call; if the energy change is 1 keV or more from the current setting, a full beamline alignment sequence is automatically inserted. Layer 4 detects empty LLM responses and retries once with explicit guidance. Layer 5 validates energy ranges, removing actions that reference energies outside the 5–25 keV operating window. Layer 6 enforces domain rules: function signature validation, motor group constraints (*e.g.* homing commands restricted to KOHZU stages), and scan–motor compatibility checks. Layer 7 implements a sample preparation gate that, in real hardware mode, blocks measurement actions (quickXanes, quickXafs, quickRaster) until the user explicitly confirms that a physical sample is mounted and aligned on the stage; this prevents empty-sample scans that would waste beam time in production operation. The complete pipeline is deterministic and stateless—each layer operates on the action list independently, enabling straightforward debugging and rule addition; the pipeline is illustrated in Fig. 5 ▸ (Section S4).

### 4.2. Multilingual validation, expert review and safeguards

A significant practical advantage of the NLP interface is its ability to lower language barriers at international user facilities. We validated the action-identification accuracy across three languages—Korean, English, and Japanese—achieving 98.2% action-identification accuracy (228 test cases across 44 categories). Preliminary tests with seven additional languages (Chinese, Arabic, Hindi, German, French, Thai, Spanish) confirmed that the underlying LLMs understand all tested languages (100% comprehension), but the current prompt design limits action-identification accuracy for these languages. The browser’s 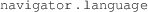 API (WHATWG, 2024 ▸) automatically selects the user’s preferred language at first load. For example, the Korean request[Chem scheme1]
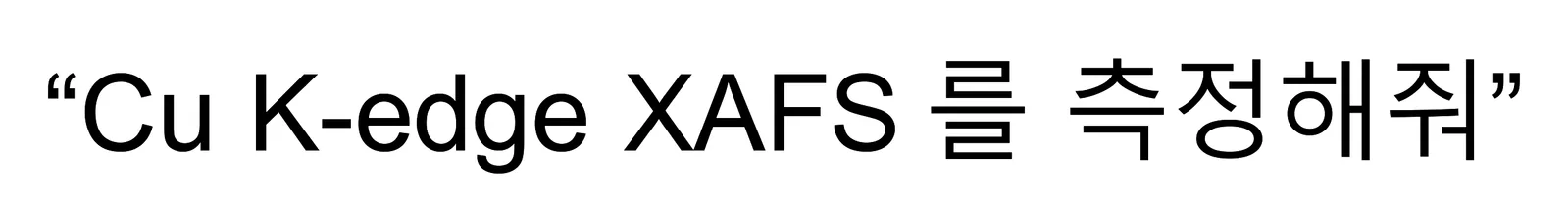
and the Japanese equivalent[Chem scheme2]
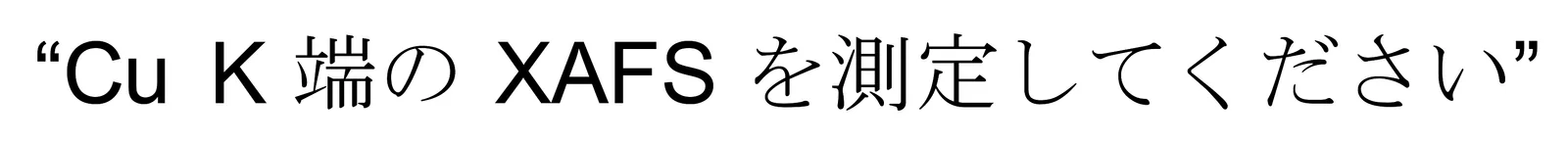
(both meaning ‘measure the Cu *K*-edge XAFS’) both produce identical structured actions. Extending prompt templates with multilingual few-shot examples is a straightforward engineering task addressed as ongoing Phase 1 development.

Through three rounds of systematic prompt engineering (Table S14), the 8 B model’s accuracy rose from 56.1% to 89.6% (on the contemporaneous 82- and 154-test suites), a larger gain than scaling to 32 B provides (+8.5 pp at the v2.0 prompt, where the 235 B MoE model does not improve further at 92.9%). This demonstrates that domain-specific prompt refinement can be more cost effective than model scaling for structured output tasks. We evaluated the NLP interface using a benchmark test suite of 228 core prompts spanning 44 categories including basic motor control, scans, alignment, multi-element experiments, edge cases, rejection of out-of-range requests, and virtual experiment workflows, plus 35 multilingual prompts covering the seven additional languages. Each prompt was evaluated for correct action identification, parameter extraction, and safety compliance. Table 3 ▸ summarizes the results across five model configurations: the local Qwen3 family at three sizes (8 B, 32 B, and 235 B MoE) across the Ollama and vLLM engines, together with the Solar Pro3 cloud model. These were chosen to isolate the effects of model size and serving engine on a fixed prompt; the remaining hosted backends (Gemini, Groq, and Claude) are integrated for redundancy but were not part of this controlled comparison. The recommended production configuration—Qwen3-32B on vLLM with prefix caching—achieves 98.2% pass rate (224/228) with 21.6 s average latency on dual NVIDIA A6000 GPUs. Solar Pro3, a Korean-optimized cloud model, achieves 94.7% at 2.9 s average latency, demonstrating that cloud APIs can serve as viable alternatives when local GPU resources are unavailable.

We further validated the system through expert review. Two beamline scientists with operational experience at third-generation synchrotron facilities independently evaluated all 228 automated test responses. Each response was classified as OK (the generated actions would produce a correct and complete experimental result on a real beamline) or Not-OK (missing parameters, suboptimal choices, or safety concerns that an experienced operator would not accept). The expert satisfaction rate was 67.3% (average 153.5/228 OK), significantly lower than the automated pass rate of 98.2%. We regard this 30.9 percentage-point gap between automated correctness and expert acceptance as the central practical finding of the NLP evaluation: automated structural correctness does not equate to operational acceptability, and reaching deployment-grade natural-language control requires closing this gap rather than optimizing the automated metric alone. We provide a quantitative decomposition of this gap from the two experts’ per-response verdicts (Section S5.1 and Table S17): the inter-expert agreement is 66.2% (Cohen’s kappa 0.23), and, when each Not-OK judgment is attributed according to whether the other expert accepted the same response, then operator-preference ambiguity accounts for 52% of the rejections (an upper bound, since inter-expert disagreement also captures cases where one expert simply applies a stricter standard) while genuine deficiencies that both experts reject account for the remaining 48%, so operator-preference ambiguity is a major but not the sole contributor to the gap. For example, an instruction such as ‘set it up for Fe XAFS’ is interpreted by one operator as staging the parameters only, while another expects the scan to be queued and started immediately; whichever interpretation the agent chooses, the other reviewer tends to mark the response Not-OK. Similarly, a request to ‘measure the map densely’ may be realized by reducing the step size to the beam-size scale or, equivalently, by increasing the number of points over the same field of view, and different operators prefer different resolutions of this ambiguity. Categories such as exposure time specification (25 cases), energy-dependent alignment criteria (12 cases), scan parameter negotiation (10 cases), and multi-element XRF energy selection (8 cases) do not have uniquely correct answers but depend on operator preference and experience.

Recognizing this characteristic, we are pursuing prompt and post-processing improvements that involve a deliberate choice between two tuning strategies. The first personalizes prompts and post-processing rules to the preferences of a specific beamline manager or operator. The second establishes consensus-based sensible defaults and introduces an interactive clarification step that asks the user to disambiguate requests whose intent is inherently under-specified. Our current system adopts the latter as the default path while being designed so that a per-facility personalization layer can be optionally overlaid on top. Because the same request can be operationally correct for one operator and not for another, a multi-user production deployment would also require this personalization to become a per-user layer rather than a single shared configuration, alongside the multi-user queue, data-access and authentication infrastructure that the operational phase will introduce. The expert insights above have been systematically categorized into ten priority improvement areas (Section S5) and are being incorporated into the system prompt and post-processing pipeline accordingly.

The four automated test failures (4/228 = 1.8%) were analyzed individually. Two occurred on complex multi-step requests—a sequential two-edge X-ray absorption near-edge structure (XANES) workflow (Ti then Sr *K*-edge on an SrTiO_3_ sample) and a 2D X-ray-fluorescence mapping experiment-preset setup—for which the agent returned an empty action list instead of the expected multi-step plan. One occurred because the agent chose a direct raster scan rather than the expected beamline-optimization and signal-estimation step for a trace-contamination query (copper at ∼10 p.p.m. in a cathode material). The fourth produced the correct optimization action but did not raise the confirmation-required flag that the reviewers expected for an ambiguous sample-preparation context. All four failure modes are addressable through additional few-shot examples together with post-processing rules for empty-response retry and confirmation-flag enforcement. The end-to-end NLP response latency, from user input to action plan display, is the 21.6 s average reported in Table 3 ▸ for the recommended vLLM Qwen3-32B backend; on the same hardware Ollama requires 67 s, and the per-backend breakdown is given in Table S16. We note that the NLP interface inherits well known LLM limitations including occasional hallucination and sensitivity to prompt phrasing. The mandatory confirmation step and structured output validation mitigate the risk of erroneous motor actions.

The current system enforces several safeguards against erroneous NLP-driven actions: mandatory user confirmation before any motor motion, deterministic schema validation of all LLM-generated actions (rejecting unknown function names and out-of-range arguments), energy-range and motor-group constraint checks, and a sample-preparation gate that blocks measurement actions until a sample is confirmed (Layers 5–7 above). For example, absorption edges outside the 5–25 keV operating range (such as Si or C *K*-edges below 5 keV, or a W *K*-edge at 69.5 keV), non-physical or out-of-range energies (negative, or far outside the operating window such as 100 keV), and unavailable operations (such as optical imaging) are rejected or redirected to a feasible alternative (for instance an accessible *L*_3_ edge), while under-specified requests trigger a clarifying question rather than a guessed action and urgent stop phrasings are mapped to an immediate halt. These behaviors are covered by dedicated rejection, safety and edge-case categories in the publicly released 228-prompt benchmark (Table S15), allowing independent reproduction. For multi-user production deployment we identify the additional controls that will be required: token-based authentication and authorization for PV writes, sandboxing of agent-generated actions, and input validation against malformed or adversarial prompts. These hardening measures are planned for the operational phase, pending the facility’s security policy, and are not yet implemented.

## 5. Virtual commissioning and results

### 5.1. Alignment procedures

A key application of this platform is virtual commissioning: rehearsing the alignment procedures that will be performed on the real beamline. Five alignment procedures are implemented using the same *Bluesky* scan plans and *Ophyd* devices that will operate on the real beamline. In the M1 and M2 half-cut procedure, a blade is scanned through the beam and the absolute derivative of the transmitted intensity is fitted with a Gaussian to locate the beam center and width. The DCM fine-pitch rocking scans the second-crystal pitch and fits the SSA-transmitted flux with a Gaussian to locate the operating angle. Mirror pitch optimization scans the M1 or M2 grazing angle and fits the downstream flux with a Gaussian to find the optimum, and SSA centering uses dual Gaussian fitting on the positive and negative derivative peaks of a blade scan through the secondary source aperture. Kirkpatrick–Baez nano-focusing requires particularly fine adjustment: with extreme demagnification ratios of 296:1 for KB-V (*p* = 91.69 m, *q* = 0.31 m) and 919:1 for KB-H (*p* = 91.90 m, *q* = 0.10 m), a pitch deviation of only a few microradians from the optimal 3 mrad grazing angle produces measurable beam-size degradation at the sample. The optimization scans KB-V pitch over a ±0.5 mrad range in 21 steps while recording the beam FWHM at the sample plane via the Monte Carlo engine, and a Gaussian fit identifies the minimum; the procedure is then repeated for KB-H. Because KB-V affects only the vertical focus and KB-H only the horizontal, the two optimizations are independent and converge in a single pass. All fits report *R*^2^ quality metrics with automatic fallback to centroid-based analysis when the fit quality is poor (*R*^2^ < 0.8), and the complete automated sequence proceeds as M1 pitch, DCM rocking, M2 pitch, SSA centering, and KB-V/H optimization. Because these algorithms use the same scan plans and *Ophyd* devices as production operation, the procedures developed during virtual commissioning transfer directly to the real hardware without modification at the validated device layers (Section 2); this is valuable for KB alignment, which on a real nanoprobe beamline typically requires hours of iterative manual adjustment.

### 5.2. Full-stack demonstration

**Figure 6:**
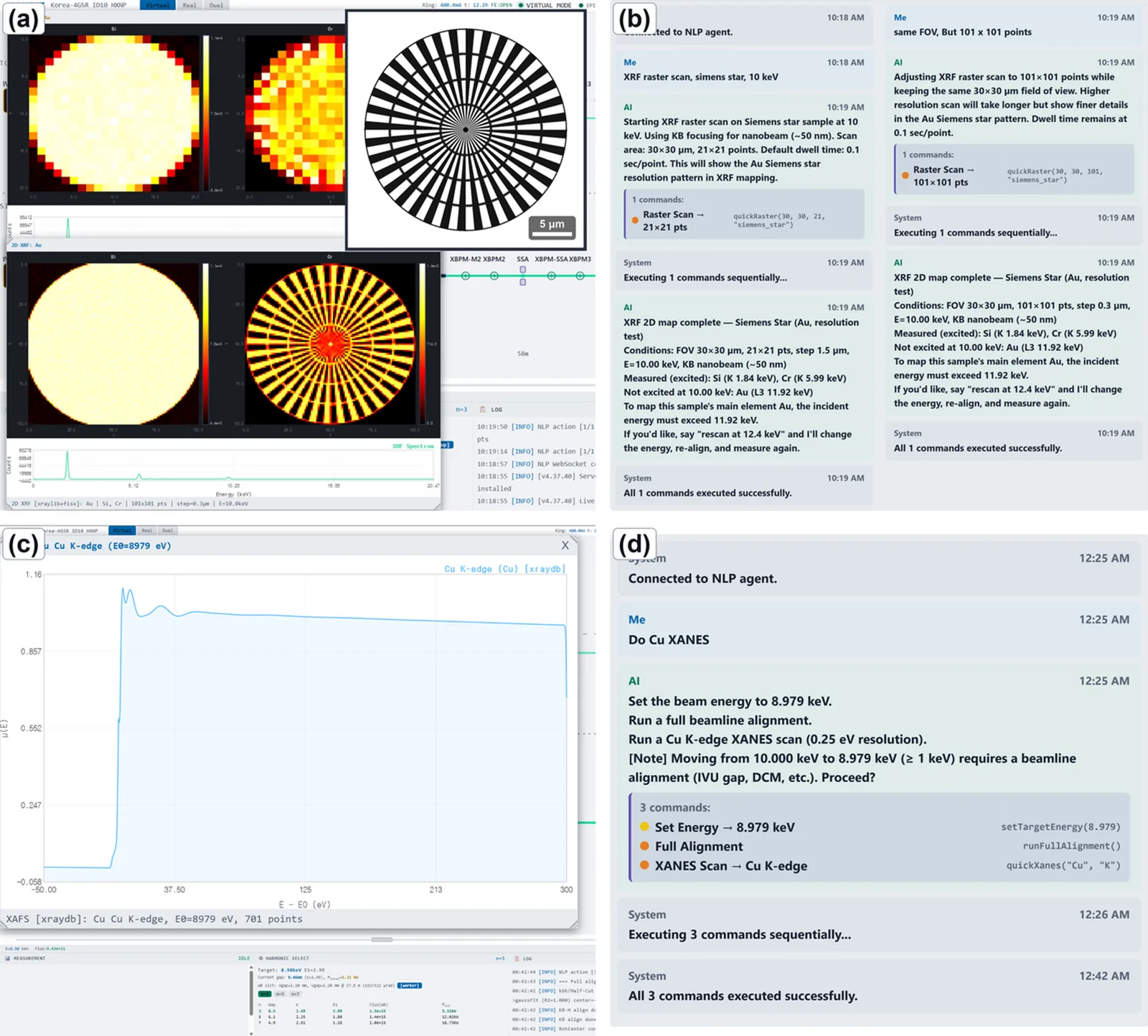
NLP-driven virtual experiments captured directly from the browser client. (*a*) Element-resolved XRF maps from a natural-language-driven raster scan at 10 keV of a Siemens-star resolution target, modeled as a 0.5 µm Au/Cr/Si thin film (predominantly Au) and generated by the *xraylib* and *fisx* scan engine. At 10 keV the Si (*K*-edge 1.84 keV) and Cr (*K*-edge 5.99 keV) fluorescence lines are mapped, while the dominant Au is not excited (Au *L*_3_ = 11.92 keV); the radial spokes are clearly resolved at the refined 101 × 101 (10201-point) raster scan but not at the initial 21 × 21. The inset shows the reference Siemens-star spoke pattern. (*b*) NLP chat panel for the XRF raster, showing the plain-language request, issued with a deliberate misspelling (‘simens star’) that the pre-processing layer normalized to the correct Siemens-star preset, and the agent’s action list. (*c*) Cu *K*-edge XANES: the energy-change guard (d*E* ≥ 1 keV) automatically inserts a full alignment sequence (M1, DCM, M2, SSA, KB) before the energy step scan; the panel shows the simulated μ(*E*) spectrum (701 points) across the Cu *K*-edge (*E*_0_ = 8979 eV). (*d*) NLP chat panel for the Cu *K*-edge XANES, showing the plain-language request and the agent’s action list.

**Figure 7:**
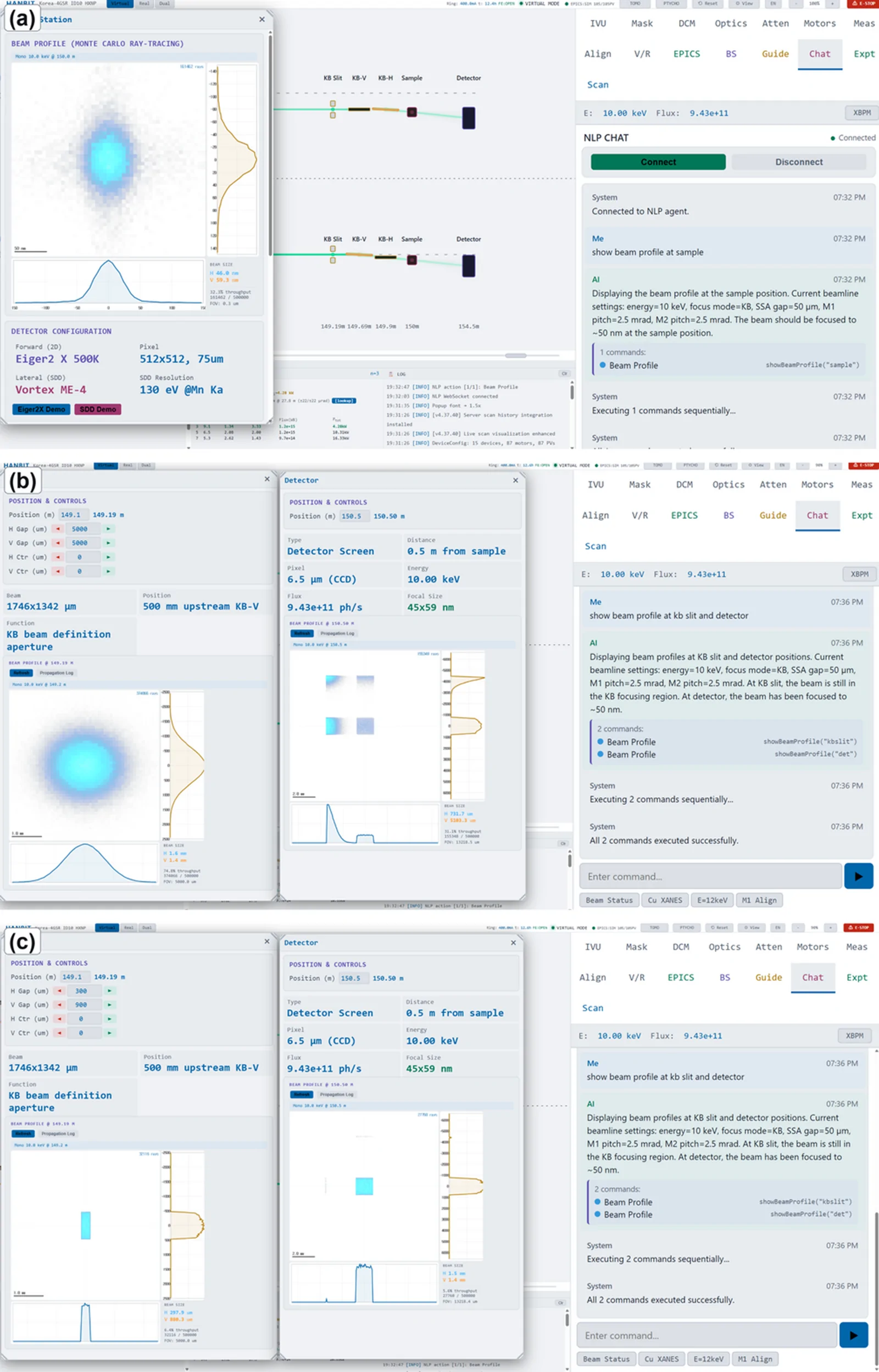
Kirkpatrick–Baez nano-focusing alignment captured from the browser client, demonstrating that the Monte Carlo optics engine reproduces the slit-clipped beam shapes used in practice to align the KB mirror pair. (*a*) Focused-beam profile pop-up panel at the sample plane showing the fully aligned state: the 2D spot is displayed with horizontal and vertical 1D cuts and the measured FWHM values, showing the simulated focus obtained after the automated alignment sequence has converged. (*b*, *c*) Two intermediate states of a KB beam-defining slit scan: the slit aperture just upstream of the KB pair is varied and the resulting beam pattern observed on the downstream detector screen changes accordingly. When the slit is opened wide (*b*), four approximately square distributions appear on the detector screen: the doubly reflected focused beam (reflected by both KB-V and KB-H), the direct bypass beam (reflected by neither mirror), and the two singly reflected beams (KB-H only and KB-V only). When the slit is closed down (*c*), it selects the doubly reflected focused beam and the pattern is a single distribution.

We demonstrate the beamline system operating in full-stack mode: the browser client communicates via WebSocket with the Python server, which connects to the *caproto* soft IOC via Channel Access, and *Bluesky* executes scan plans through *Ophyd* EpicsMotor devices. Fig. 6 ▸ shows two representative NLP-driven virtual experiments captured directly from the browser client. In (*a*), an XRF raster scan of a Siemens-star resolution target at 10 keV is requested through a short, deliberately misspelled prompt (‘simens star, 10 keV’), which the pre-processing layer normalizes to the correct Siemens-star preset before the agent dispatches the scan. The target is modeled as a 0.5 µm Au/Cr/Si thin film, so at 10 keV only the Cr and Si *K*-lines are mapped while the dominant Au remains unexcited (Au *L*_3_ = 11.92 keV). An initial 21 × 21 raster does not resolve the radial spokes; a follow-up natural-language request to keep the same field of view at 101 × 101 points refines the scan until the spokes are clearly resolved, demonstrating interactive parameter refinement through dialog. In (*c*), a Cu *K*-edge XANES scan is requested; because the energy move from 10 keV to the 8.979 keV edge exceeds the 1 keV change threshold, the guard automatically inserts a full alignment sequence before the multi-region step scan. Panels (*b*) and (*d*) show the corresponding NLP chat panels, with the plain-language requests and the agent’s generated action lists for the raster scan (*b*) and the XANES scan (*d*). In both cases the agent proceeds from natural-language input to a completed measurement result without manual configuration. Fig. 7 ▸ illustrates that the same virtual platform also reproduces the physical alignment behavior of a real nano­probe beamline: (*a*) the focused-beam profile pop-up panel at the sample plane after the KB-V and KB-H pitches have been optimized, and (*b*, *c*) two intermediate states of a KB beam-defining slit scan in which the slit aperture is varied and the resulting beam pattern observed on the downstream detector screen changes accordingly, demonstrating that the Monte Carlo engine captures the slit-clipped beam shape used in practice to align the KB mirror pair. The complete workflow, from a Korean-language knowledge query through alignment, XAFS and XRF scans to a final switch of the platform into real hardware mode, is also provided as a continuous screen recording (Movie S1); the step-by-step session is described in Section S9.

### 5.3. Performance and latency

**Figure 8:**
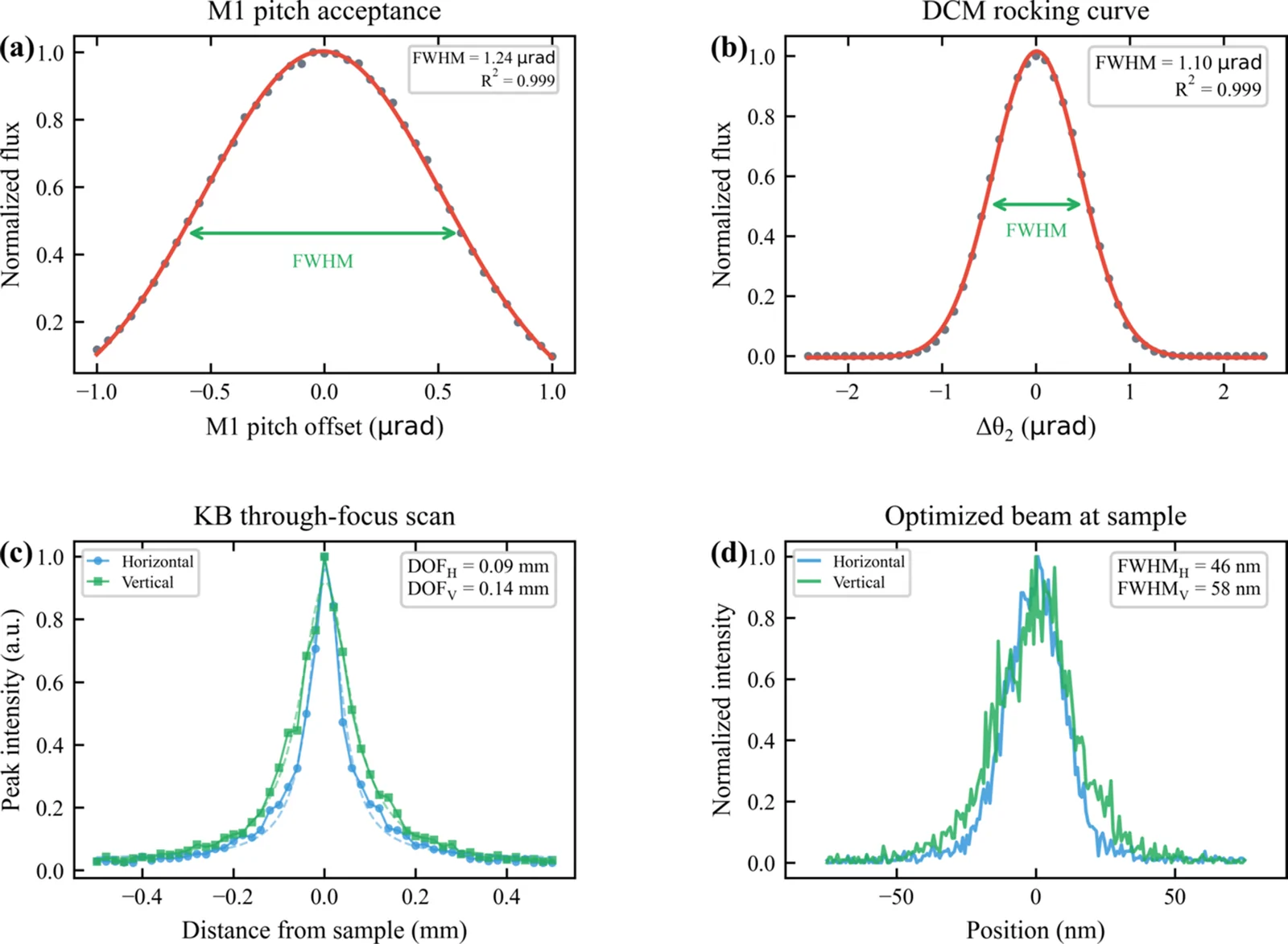
Virtual commissioning alignment and characterization results obtained with the same *Bluesky* scan plans and *Ophyd* devices that will operate the real beamline. (*a*) M1 pitch acceptance: the M1 grazing pitch is scanned about its 2.5 mrad operating angle and the SSA-transmitted flux is fitted with a Gaussian (FWHM = 1.24 µrad, *R*^2^ = 0.999), quantifying the angular tolerance of the M1 alignment. (*b*) DCM Si(111) second-crystal rocking: the second-crystal fine pitch (Δθ_2_) is scanned about its operating angle and the transmitted flux is fitted with a Gaussian (FWHM = 1.10 µrad, *R*^2^ = 0.999), quantifying the angular tolerance of the DCM second-crystal alignment. (*c*) KB through-focus scan: the peak beam intensity is recorded as the evaluation plane is stepped through the sample position, and a Lorentzian fit gives a depth of focus (DOF) of 0.09 mm horizontally and 0.14 mm vertically. (*d*) Optimized beam at the sample plane: one-dimensional horizontal and vertical intensity profiles of the fully aligned focus, with FWHM = 46 nm (horizontal) and 58 nm (vertical). The microradian-scale acceptance widths in (*a*) and (*b*) are set by the beam walk across the 50 µm SSA over the 27.6–29 m lever arms (aperture-geometry-limited), not by the intrinsic mirror or crystal angular acceptance.

The five virtual commissioning procedures above were executed end-to-end and produced high-quality fits throughout, with the complete automated sequence running to completion without manual intervention. Fig. 8 ▸ shows representative results: the M1 pitch acceptance fitted to a Gaussian with FWHM = 1.24 µrad and *R*^2^ = 0.999 [Fig. 8 ▸(*a*)]; the DCM second-crystal fine-pitch acceptance fitted to a Gaussian with FWHM = 1.10 µrad and *R*^2^ = 0.999 [Fig. 8 ▸(*b*)]; a KB through-focus scan yielding a depth of focus of 0.09 mm (horizontal) and 0.14 mm (vertical) [Fig. 8 ▸ (*c*)]; and the optimized focus at the sample plane with FWHM = 46 nm (horizontal) and 58 nm (vertical) [Fig. 8 ▸(*d*)]. System performance was characterized under realistic load: the measured 51-point energy scan (9.9 to 10.1 keV) completed in 7.8 s, with a mean per-event overhead of 0.4 ms for event serialization and WebSocket transport [about 21 ms across the 53 event documents, *i.e.* the 51 scan points plus two baseline readings (Section S1.2), 0.27% of the scan]; for a production scan with 1 s per-point integration (about 58 s total) this transport overhead falls below 0.05%. Table 4 ▸ reports end-to-end latencies for two measurement paths (illustrated in Fig. S2): a localhost software floor, where server and client are co-located on the same host so the figures characterize serialization overhead rather than network performance and are bounded below by timer resolution, and a realistic remote path, where the client reads from the server over a routed network with Network Address Translation (NAT). On the remote path the PV-monitoring write-to-client round-trip, which is single-clock measurable directly, has a median of 91 ms, dominated by the server’s 100 ms PV broadcast loop, versus approximately 30 ms on the localhost floor. Because the client and server do not share an NTP-synchronized clock, the remote one-way scan and WebSocket-transport latencies are not directly measurable; they are estimated by adding a cross-host clock offset derived from a single-clock TCP round-trip probe (median 2.4 ms, implying about 1.2 ms one-way transit), giving a remote one-way scan-event median of 2.3 ms. These one-way figures reflect the serialization floor plus the network transit (not the transit alone), and are therefore reported as estimates rather than operational transport values. These values are comparable with established production systems (Allan *et al.*, 2019*a* ▸; Yu *et al.*, 2024 ▸) and remain well within motor-speed control requirements (Sections S1 and S6). The measurement script is released publicly as tools/measure_latency.py.

### 5.4. Virtual experiment toolkit

The virtual experiments shown in Fig. 6 ▸ are driven by a set of simulation tools that the NLP agent can invoke through the standard action interface, all executing entirely within the browser client alongside the Monte Carlo optics engine. A virtual XRF detector computes element-resolved fluorescence line intensities on the fly using *xraylib* (Schoonjans *et al.*, 2011 ▸) and *fisx* (the C++ port of the *PyMca* physics routines; Solé *et al.*, 2007 ▸; https://github.com/vasole/fisx) cross-sections, with energy-dependent absorption, self-absorption, and a simple detector-efficiency model; the same engine produces the element-resolved raster maps shown in Fig. 6 ▸(*a*). A virtual XANES/XAFS generator produces simulated X-ray absorption coefficient μ(*E*) spectra across the 5–25 keV operating window, using pre-tabulated edge energies and white-line features for elements from Ti to Pb, and is the source of the spectrum shown in Fig. 6 ▸(*c*). Both the virtual XRF detector and the XANES/XAFS generator consume the beam size and photon flux delivered by the Monte Carlo optics engine at the current beamline state, so that their signal levels, count-rate statistics and spot-sensitive features respond automatically to any change in energy, mirror coating, SSA aperture, or KB alignment that the user makes in the browser client. Virtual ion chambers and beam-position monitors provide realistic transmission and position readouts so that the same *Bluesky* scan plans and *Ophyd* devices used during virtual commissioning consume identical signal streams on the real beamline. A virtual sample library provides pre-defined geometries including the Siemens-star resolution target shown in Fig. 6 ▸(*a*), as well as representative battery cathode materials [lithium nickel manganese cobalt oxide (NMC), LiFePO_4_], thin-film reference standards, and biological test objects, each with its own element distribution and morphology; users can also upload custom sample definitions through the NLP chat interface. Acquired datasets are written through the standard NeXus/HDF5 and SQLite pipeline described in Section 2. Together, this toolkit allows a natural-language request to produce a fully simulated but scientifically meaningful measurement result within seconds, giving users a realistic preview of an experiment before any real beam time is consumed.

Beyond experiment preview, the simulation results help prepare the commissioning protocol and operational parameters in advance of real-beam time. During virtual commissioning, the KB pitch-scan procedure confirmed that the vertical focus is more sensitive to pitch errors than the horizontal focus; this observation has been incorporated into the commissioning protocol as a tighter pitch tolerance for KB-V. The SSA aperture sweep indicated that reducing the aperture below 30 µm provides diminishing spatial-resolution gains while incurring disproportionate flux loss, supporting 50 µm as a practical default for general nano-XRF operation. These parameters were established in advance of optical-component installation, illustrating that the virtual platform serves not merely as a training tool but as a quantitative aid for commissioning preparation, complementing rather than replacing the established beamline design.

## 6. Summary and outlook

We have presented a virtual commissioning platform that enables beamline scientists to design, test and refine the complete operational workflow of the KLS ID10 Hard X-ray Nanoprobe beamline years before the first photon arrives. The platform provides three capabilities that are not available from any single existing tool: real-time Monte Carlo optics simulation in the browser for interactive parameter exploration, standard *EPICS* and *Bluesky* control integration so that scan plans and *Ophyd* device definitions developed virtually transfer unchanged to the real beamline at the validated device layers, and a multilingual natural-language interface that allows non-specialist users to operate the instrument in their native language. We characterize this system as a pre-installation virtual commissioning platform rather than an operational digital twin, using the latter term only in the broad sense of Grieves & Vickers (2017 ▸) [see also Tao *et al.* (2019 ▸; Jones *et al.* (2020 ▸)], and we regard full digital-twin functionality, including bidirectional state synchronization with operating hardware, as a future goal to be realized once the physical beamline exists.

The Monte Carlo engine is validated against *SPECTRA* undulator spectra, source size and divergence (energy spread and emittance included) and reproduces the overall shape of the *Shadow4* focused-beam profiles, the NLP agent reaches 98.2% automated action-identification accuracy with expert review identifying a 30.9 percentage-point gap to operational acceptability as the central remaining challenge, and the zero-change hardware transition strategy has been validated with three real subsystems.

Looking ahead, the remaining optical subsystems (IVU, DCM, M1/M2, KB) will be progressively integrated as components are installed during 2027–2029, and each integration milestone will generate a new round of simulation-informed design feedback. The server’s monitoring path will move from the periodic PV broadcast described in Section 2 to event-triggered push, which delivers changes immediately while coalescing rapid bursts to keep the message rate bounded, and the NLP agent will continue to incorporate expert feedback through beamline-manager personalization and consensus-based defaults with interactive clarification. By the time real photons arrive at ID10 for first light in 2029, the alignment procedures, scan plans, and NLP-driven workflows will have undergone three years of iterative refinement, which is intended to shorten the on-site commissioning of Korea’s first fourth-generation hard X-ray nanoprobe beamline, a phase that would otherwise consume scarce early beam time. We anticipate that the approach demonstrated here, combining browser-based simulation with standard control-system integration and natural-language operation, is transferable to other beamlines at KLS and at fourth-generation facilities worldwide.

## Supplementary Material

Supporting Sections S1 to S9. DOI: 10.1107/S1600577526006788/ok5168sup1.pdf

Demonstration movie. DOI: 10.1107/S1600577526006788/ok5168sup2.mp4

## Figures and Tables

**Table 1:** Comparison with related systems

Feature	This work	*Mamba* (HEPS)	BESSY II DT	*Daiquiri* (ESRF)
Ray tracing	• (browser)	–	• (*RAY-UI*)	–
*EPICS* integration	•	•	•	– (*TANGO*)
Scan engine	•	•	•	–
Web-based UI	•	– (*Qt desktop*)	–	•
NLP control	•	•†	–	–
Virtual commissioning	•	–	•	–

† HEPS LLM agent reported at NOBUGS 2024 (Bi *et al.*, 2024 ▸).

**Table 2:** Undulator harmonic comparison (this work versus *SPECTRA*) at the representative operating point *K* = 1.87 (gap 7.14 mm), where harmonics *n* = 3–11 span roughly 5–25 keV Listed are the on-axis angular flux density, a far-field quantity independent of distance from the source, and the flux through the white-beam slit (1.2 mm × 1.2 mm at 27.8 m from the undulator center, ±21.58 µrad), obtained by integrating the angular flux density over that acceptance. Deviations are computed from unrounded values.

Harmonic	Energy (keV)	Flux density, this work [photons s^−1^ mrad^−2^ (0.1% bandwidth)^−1^]	Flux density, *SPECTRA* [photons s^−1^ mrad^−2^ (0.1% bandwidth)^−1^]	Deviation	WB-slit flux, this work [photons s^−1^ (0.1% bandwidth)^−1^]	WB-slit flux, *SPECTRA* [photons s^−1^ (0.1% bandwidth)^−1^]	Deviation
3rd	6.91	2.71 × 10^18^	2.71 × 10^18^	+0.3%	2.15 × 10^15^	2.12 × 10^15^	+1.2%
5th	11.52	1.55 × 10^18^	1.55 × 10^18^	+0.2%	9.76 × 10^14^	9.72 × 10^14^	+0.4%
7th	16.13	8.85 × 10^17^	8.81 × 10^17^	+0.4%	4.49 × 10^14^	4.49 × 10^14^	+0.1%
9th	20.73	5.04 × 10^17^	5.01 × 10^17^	+0.6%	2.14 × 10^14^	2.14 × 10^14^	+0.3%
11th	25.34	2.86 × 10^17^	2.84 × 10^17^	+0.7%	1.11 × 10^14^	1.10 × 10^14^	+1.3%

**Table 3:** NLP action-identification accuracy by model and engine (228 core + 35 multilingual test cases, 44 categories)

Model	Engine	Tests	Pass rate	Average latency
Qwen3-32B	vLLM (local, 2xA6000)	228	98.2%	21.6 s
Qwen3-32B	Ollama (local)	154†	98.1%	67.0 s
Solar Pro3	Cloud API	228	94.7%	2.9 s
Qwen3-8B	Ollama (local)	154†	89.6%	35.7 s
Qwen3-235B MoE	Ollama (local, Q3)	154†	92.9%	163.4 s

† 154 tests (v2.0 prompt, 28 categories).

**Table 4:** End-to-end latency budget for the real-time data pipeline, measured for two paths: a localhost software floor (server and client on the same host, bounded below by timer resolution) and a realistic Remote path (a routed network with NAT) The Remote one-way scan-event and WebSocket-transport rows are clock-offset-corrected estimates rather than operational transport values (Section 5.3; Sections S1 and S6).

Stage	Measured (localhost, software floor)	Measured (Remote, Client to Server via NAT)
Scan event median	< 1 ms	2.3 ms
Scan event mean	0.4 ms	2.3 ms
Scan event max	2.0 ms	4.8 ms
Scan event P90	1.0 ms	2.4 ms
PV: WS transport median	<1 ms	1.2 ms
PV: total median (sim)	31 ms	91 ms
PV: total mean (sim)	29 ms	93 ms
