# Supplementary material for: Virtual commissioning of the Korea Light Source Hard X-ray Nanoprobe beamline with multilingual natural language control
Source: J Synchrotron Radiat. 2026 Jul 31;33(Pt 5):1310–25. doi: 10.1107/S1600577526006788 (PMC13549346; doi:10.1107/S1600577526006788)
Supplement: Supplementary file 1 [file s-33-01310-sup1.pdf]

JOURNAL OF SYNCHROTRON RADIATION

Volume 33 (2026)

Supporting information for article:

Virtual commissioning of the Korea-4GSR Hard X-ray Nanoprobe beamline with multilingual natural language control

Minho Seo, Hyeong-joo Choi, Dasuel Ham Ham, Changwan Ha, Yong-Hyun Kim, Changhui Lee, Jun Lim, Ki-jeong Kim, In-hui Hwang and Jaeyong Shin

S1. Latency Measurement Methodology

S1.1 Instrumentation

To measure the end-to-end latency of both data pipelines, we inserted timestamp instrumentation at each stage of the pipeline:

Server-side (Python):

- _ts_callback: time.time() recorded at entry to _document_callback() (RunEngine document received).
- _ts_send: time.time() recorded immediately before the async broadcast is scheduled.
- _ts_ws_send: time.time() recorded in broadcast_scan_event() immediately before WebSocket send().
- _ts_pv_send: time.time() recorded in pv_broadcast_loop() before sending PV updates.

Client-side (Python WebSocket client):

- _ts_client_recv: time.time() recorded immediately upon WebSocket message receipt. All timestamps use Python's time.time() (Unix epoch; effective granularity about 1 ms on the measurement hosts, which sets the <1 ms reporting floor of Tables S1 and S2). In the localhost scenario the server and measurement client run on the same machine, so no clock synchronization is required; in the Remote scenario the two hosts differ and the cross-host clock offset is recovered from a single-clock TCP round-trip probe (Section S1.3).

S1.2 Measurement procedure

Scan event latency: 1. Start the caproto soft IOC (python server/epics/soft_ioc.py) 2. Start the server with Bluesky enabled (python server/server.py --bluesky) 3. Connect a WebSocket client to ws://localhost:8001/ws/scan 4. Submit a 51-point energy scan (9.9–10.1 keV) 5. Record timestamps for all 57 document events (1 start + 2 descriptors + 53 events + 1 stop; the 53 events comprise the 51 scan points plus 2 baseline readings) 6. Compute per-event latencies: total = ts_client_recv - ts_callback.

PV monitoring latency: 1. Connect a WebSocket client to ws://localhost:8001/ws/pv 2. Subscribe to BL10:DCM:DTheta2 and BL10:DCM:DTheta2.RBV 3. Write alternating values (0, 0.001) to trigger PV updates 4. Record 23 PV update events over 10 seconds 5. Compute latencies: - send_to_recv = ts_recv - ts_pv_send (WebSocket transport only) - write_to_recv = ts_recv - ts_write (total including PVStore scan/broadcast cycle)

S1.3 Measurement scenarios (localhost, Remote)

Two measurement scenarios are compared in this Supplementary, both using the same measurement script (tools/measure_latency.py) and the same HANBIT server, but differing in the network path between the client and the server (Figure S2). (a) Localhost (software floor): client and server run on the same physical host, communicating via loopback; a single Python

time.time() clock is used. The scan-event and WebSocket-transport medians are sub-millisecond and limited by its ~1 ms granularity, characterizing software-serialization overhead rather than facility-network performance, whereas the PV write-to-client median (~31 ms, Table 4) is set by the server's 100 ms broadcast loop. (b) Remote: the client runs on a separate host and reaches the server over a routed network with NAT. Because the client and server do not share a common time source (no NTP between hosts), the quantity that is single-clock measurable directly is the client-clock-only round trip, namely the PV write-to-client-receive latency (median 91 ms, dominated by the server's 100 ms PV broadcast loop). One-way scan and WebSocket-transport latencies are not single-clock measurable on this path; to report them without requiring NTP, the measurement script begins each Remote run with a single-clock TCP round-trip probe (HTTP GET against the server endpoint, 30 iterations, median 2.4 ms), takes half the median TCP Round Trip Time (RTT, about 1.2 ms) as an estimator of the one-way transit, and adds the resulting cross-host clock offset to the apparent server-to-client timestamps, yielding a one-way scan-event median of 2.3 ms. These one-way figures reflect the serialization floor plus the network transit (not the transit alone), so they are reported as estimates rather than operational transport values. The corresponding measured values are listed in main-text Table 4.

S2. Hardware Validation Details

The zero-change hardware transition strategy (Section 2) was validated with three real hardware subsystems on a test network prior to beamline installation. This section provides the technical details of each subsystem and compares the soft IOC and hardware IOC configurations.

S2.1 Test Network Topology

The test network comprises four network switches:

Switch	Subnet	Purpose
SW1 (Campus)	a.a.a.0/24	External SSH/HTTP access
SW2 (Management)	b.b.b.0/24	Virtual Machine internal management
SW3 (EPICS)	c.c.c.0/24	EPICS IOC network
SW4 (Device)	d.d.d.0/24	Direct device control

The beamline server (Virtual Machine, Ubuntu 22.04, 4 cores, 8 GB RAM) hosts the soft IOC, CA bridge, and Python server. Hardware devices connect via dedicated network interfaces.

S2.2 Soft IOC vs Hardware IOC Comparison

Table S4 compares the soft IOC simulation and real hardware IOC configurations for each validated subsystem. The key difference is that the soft IOC uses caproto FakeMotor with velocity-limited simulated move semantics, while the real IOCs connect to physical hardware with actual settling times and resolution limits. Despite these differences, the same ophyd EpicsMotor class is used in both cases, and no client code modification is required.

S2.3 Hybrid Mode Operation

In hybrid mode, the soft IOC and hardware IOC coexist on the same Channel Access network. The server's --exclude-groups flag removes hardware-served PV groups from the soft IOC (SAM for the sample stage, and analogously XBPM2 for the beam-position monitor), allowing the corresponding hardware IOC to serve those PVs instead. The browser UI displays HW/SIM badges on each motor to indicate the PV source.

PV discovery. At startup, the server performs automatic PV discovery by querying all registered PV names via Channel Access. PVs served by hardware IOCs are tagged as "HW" and trigger the safety confirmation dialog before any motor motion.

Safety layer. Before executing any motor command on a hardware-tagged PV, the system displays a confirmation dialog showing the target motor name, current position, requested position, and travel distance. The user must explicitly approve before the command is sent to the hardware IOC.

S2.4 End-to-End Verification Results

Table S5 presents the end-to-end verification results for each hardware subsystem. The motion subsystems achieved position errors within one motor resolution step (MRES) for the KOHZU axes and below 0.1 nm for the SmarAct closed loop, and the Sydor chain reproduced

the expected readback currents, confirming that the virtual-to-real transition introduces no systematic offset.

S2.5 Validated vs Remaining Subsystems

Table S6 summarizes the hardware validation coverage. Three of the fourteen device groups have been validated with real hardware: the sample stage (three axes of the 86 motor records), the beam-position monitor (12 PVs), and the nano-scanner. The first two are reached through standard EPICS motor record and analog input interfaces, and the SmarAct nano-positioner through its TCP bridge behind the same ophyd device interface. The remaining subsystems (IVU, DCM, M1/M2, KB, slits, detector, etc.) will be integrated progressively during 2027–2029 as components are installed.

S3. Physics Engine: Undulator Source, Flux Validation, and Optics Models

The browser-side Monte Carlo engine computes the IVU24 undulator source spectrum (on-axis flux density and beamline-acceptance flux) from a single-electron radiation model (Kim, 1989) convolved over the stored-beam phase space, following the same finite-emittance, finite-energy-spread treatment used by SPECTRA (Tanaka & Kitamura, 2001; Tanaka, 2014). This section describes the calculation and its numerical optimization; the resulting values are compared against SPECTRA in Table 2 of the main text and in Fig. 3. Sections S3.8 to S3.10 document the optics models used by the Monte Carlo ray-tracing engine of Section 3 of the main text: the Si(111) Darwin width, the displayed detector signal, and the focused-spot construction.

S3.1 On-axis angular flux density

For a planar undulator with N periods and deflection parameter K, the n-th odd-harmonic on-axis angular flux density is given by Eq. (S1), in photons s^{-1} $mrad^{-2}$ (0.1% bandwidth)$^{-1}$, where E and I are the electron energy and current. $F_n(K)$ is the Kim harmonic coupling function given by Eq. (S2), with argument $nK^2/(4+2K^2)$ and Bessel orders a = floor((n-1)/2), b = floor((n+1)/2). $S(K,n)$ is a suppression factor ($0 < S \le 1$) that accounts for the finite electron-beam angular divergence and relative energy spread, described next. The leading coefficient 1.744×10^{14} is the Kim (1989) flux-density constant, and J_a and J_b are Bessel functions of the first kind.

$$F_0(K,n) \;=\; 1.744 \;\times\; 10^{14}\, N^2\, E^2[\mathrm{GeV}]\, I[\mathrm{A}]\, F_n(K)\, S(K,n) \tag{S1}$$

$$F_n(K) \;=\; \frac{n^2K^2(J_a - J_b)^2}{\left(1+\frac{K^2}{2}\right)^2} \tag{S2}$$

S3.2 Energy-spread and emittance suppression

$S(K,n)$ is evaluated by numerically convolving the single-electron harmonic over the stored-beam phase space. Each electron, with angular offset (θ'_x, θ'_y) from the axis and relative energy offset δ, radiates its n-th harmonic at a red-shifted energy given by Eq. (S3); the on-axis observer therefore samples a sinc^2 line-shape displaced from resonance. Off-axis electrons red-shift the harmonic (a one-sided shift) while energy spread broadens it symmetrically, and both suppress the on-axis flux density of high harmonics. The angular dependence of the emission amplitude follows the SPECTRA far-field formulation (Tanaka & Kitamura, 2001), evaluated at each observation angle: on axis it reduces exactly to $F_n(K)$, and off axis it falls below $F_n(K)$, which suppresses the emittance red-shift shoulder. Using a

constant amplitude (which lacks this angular dependence) over-estimates high harmonics by up to about 17% at $n = 11$; including the angle-dependent amplitude removes this excess, with the resulting agreement quantified in Section S3.5. Here $E_n = nE_1$ is the on-axis resonance energy of harmonic n (E_1 the fundamental), γ the electron Lorentz factor, and θ the observation polar angle from the undulator axis. Collecting these elements, $S(K,n)$ is given by Eq. (S4), where $\rho_e(\theta'_x, \theta'_y, \delta)$ is the normalized stored-beam phase-space density, $F_n(K; \theta'_x, \theta'_y)$ is the angle-dependent emission amplitude described above (equal to $F_n(K)$ on axis), $\theta'^2 = \theta'^2_x + \theta'^2_y$ and sinc $x = (\sin x)/x$; $S \to 1$ in the zero-emittance, zero-energy-spread limit.

$$E = \frac{E_n(1+\delta)^2}{1 + \frac{\gamma^2\theta^2}{1+\frac{K^2}{2}}} \quad \text{(S3)}$$

$$S(K,n) = \int\int d\theta_x'\, d\theta_y' \int d\delta\, \rho_e(\theta_x', \theta_y', \delta)\, \frac{F_n(K;\theta_x', \theta_y')}{F_n(K)}\, sinc^2[\pi n N(\frac{\gamma^2\theta'^2}{1+K^2/2} - 2\delta)] \quad \text{(S4)}$$

S3.3 Beamline-acceptance flux

The beamline-acceptance flux is the angular flux density integrated over the acceptance defined by the beamline white-beam slit, which is located 27.8 m from the source (the IVU24 undulator center) at its 1.2 × 1.2 mm operating gap (a symmetric half-angle of ±21.58 μrad). The full opening corresponds to about $4\sigma'$ of the central radiation cone (whose RMS divergence is $\sigma' \approx 10$ μrad; over the 5–25 keV operating range the per-harmonic divergence is smaller, with a median of about 7 μrad) and is consistent with the geometric acceptance of the 600 mm mirrors of the Korea Light Source (KLS; formerly Korea-4GSR). The integral is not evaluated at a single fixed energy: because off-axis rays within the aperture are red-shifted, a fixed-energy integration omits their contribution and under-estimates the acceptance flux by 25–42%. Instead, a partial-flux spectrum is built by integrating the angle-dependent flux density (angle-dependent amplitude, red-shift factor and sinc^2 line-shape) over the slit aperture and over the electron-beam divergence and energy spread at every energy; the peak of this partial-flux spectrum (slightly red-shifted from the on-axis resonance) is taken as the acceptance flux, the same quantity evaluated by the SPECTRA partial-flux calculation through the same slit aperture (validation in Section S3.5).

This default opening also keeps the absorbed slit power within the white-beam slit's thermal envelope at the minimum undulator gap of 5 mm.

S3.4 Numerical sampling and optimization

The phase-space convolution is optimized so that every harmonic is resolved at minimum cost. Gaussian-weighted quadrature nodes are placed on $\pm 5\sigma$ of the angular divergence and of the energy spread. Grid sizes are auto-adapted per harmonic to satisfy Nyquist sampling: the angular grid spans the e-beam divergence in units of the natural (diffraction-limited) divergence $\sigma_0 = (\lambda_n/2L)^{1/2}$, and the energy grid is refined as n grows because the energy-spread sinc^2 oscillates with period $1/(2nN)$ in δ. The per-harmonic suppression factor $S(K,n)$ is cached so repeated spectral evaluations (for example, while the user sweeps the undulator gap) reuse it. The same integrator drives the interactive harmonic-selection panel, where the acceptance is set by the current white-beam-slit opening (default $H_{gap} = V_{gap} = 1.2$ mm at 27.8 m, i.e. ±21.58 μrad) rather than a fixed angle. Here $\lambda_n = hc/E_n$ is the on-axis wavelength of harmonic n and $L = N\lambda_u$ the undulator length.

S3.5 Validation against SPECTRA

The engine was validated against SPECTRA with matched source parameters (4.0 GeV, 400 mA, natural emittance 68.2 pm rad at 0.1 coupling, energy spread 1.20×10^{-3}, dispersion-free straight; λ_u = 24 mm, 123 periods (2.952 m magnetic length)). Over K = 1.0–2.5 and harmonics n = 1–15 the on-axis flux density and brilliance agree with SPECTRA to within 1% up to n = 11 (1.1% maximum over the full n = 1–15, K = 1.0–2.5 grid; Fig. S3). At the representative operating point K = 1.87, for the odd harmonics across roughly 5–25 keV (n = 3, 5, 7, 9, 11, spanning 6.91–25.34 keV) the on-axis flux density agrees to ≤0.7% and the white-beam-slit acceptance flux (1.2 × 1.2 mm at 27.8 m, ±21.58 μrad) to ≤1.3% (Table 2 of the main text; Fig. 3). The SPECTRA reference partial-flux values were computed through the same 1.2 × 1.2 mm aperture, and the SPECTRA flux density was decomposed with the zero-emittance and zero-energy-spread toggles to isolate each contribution, so that the comparison is made mode-by-mode rather than only on the fully convolved result. The validation envelope reported here covers K = 1.0–2.5 and harmonics n = 3, 5, 7, 9, 11 (the operating regime of this work); the deployed engine also ships a precomputed lookup table extending to K = 0.20–2.75 for off-design exploration, served through the lookup layer of Section S3.6 (tagged [lookup] in the user interface); the portions below K = 1.0 and above K = 2.5 lie outside the SPECTRA-validated envelope of this work.

The undulator source is characterized by the standard effective-Gaussian source size and divergence. The natural single-electron RMS size and divergence σ_r and σ'_r (Eqs. (S5) and (S6); the latter equal to σ_0 of Section S3.4) are added in quadrature with the electron-beam size and divergence, $(\varepsilon_{x,y}\beta_{x,y})^{1/2}$ and $(\varepsilon_{x,y}/\beta_{x,y})^{1/2}$, where $\varepsilon_{x,y}$ and $\beta_{x,y}$ are the horizontal and vertical electron-beam emittance and beta function at the source. In the divergence, σ'_r additionally includes the broadening by the relative electron-beam energy spread σ_E: as in SPECTRA (Tanaka & Kitamura, 2001; Tanaka, 2021), the harmonic sinc^2 line shape is convolved with the electron-energy distribution and σ'_r is replaced by the Gaussian-equivalent RMS of the resulting angular profile, equivalent to the universal-function treatment of the energy-spread effect. The correction grows with $nN\sigma_E$ (from 1.13 at the fundamental to 3.4 at n = 15) and acts identically in both planes; following SPECTRA's treatment it is applied to the divergence only (the analogous broadening of the source size stays below 4% even at n = 15 and is not applied). The resulting total RMS source size and divergence $\Sigma_{x,y}$ and $\Sigma'_{x,y}$ are listed in Tables S11 and S12 and plotted in Fig. S3(b, c), and this is the distribution from which the Monte Carlo ray tracer samples the source phase space. The values agree with SPECTRA's per-harmonic source size and divergence to within 0.6% over n = 1–15 and K = 1.0–2.5 (within 0.3% for n ≥ 3).

$$\sigma_r = \frac{\sqrt{2L\lambda_n}}{4\pi}, \quad \Sigma_{x,y} = \sqrt{{\sigma_r}^2 + \varepsilon_{x,y}\beta_{x,y}} \tag{S5}$$

$$\sigma'_r = \sqrt{\frac{\lambda_n}{2L}}, \quad \Sigma'_{x,y} = \sqrt{{\sigma'_r}^2 + \frac{\varepsilon_{x,y}}{\beta_{x,y}}} \tag{S6}$$

S3.6 Interactive computing architecture: GPU acceleration and fallback

The beamline-acceptance flux of Section S3.3 is, written out, a five-fold integral over the white-beam-slit observation angles (θ_x, θ_y; outer two dimensions), the stored-beam angular divergence (θ'_x, θ'_y; middle two dimensions) and the relative energy spread (δ; inner

dimension), with the angle-dependent amplitude of Section S3.2 evaluated at every node, as given by Eq. (S7). Here $\rho_e(\theta'_x, \theta'_y, \delta)$ is the normalized stored-beam phase-space density. The sinc^2 factor is the N-period spectral line-shape evaluated at the detuning of the photon energy E from the red-shifted resonance energy E_{res} given by Eq. (S3). In pure single-threaded JavaScript one (K, n) evaluation costs 5–30 s, so a full eight-harmonic panel would freeze the interface for minutes. To keep the harmonic-selection panel interactive while preserving the validated accuracy, the engine uses a four-layer compute cascade (js/optics/01_undulator.js, findHarmonicsAsync), each layer tagged in the user interface; its instant first response is the closed-form estimate of Eq. (S8) below, and the four layers are summarized in the following table:

$$F_{partial}(E;\ K,n) = \int\int_{slit} \mathrm{d}\theta_x\, \mathrm{d}\theta_y \int\int \mathrm{d}\theta'_x\, \mathrm{d}\theta'_y \int \mathrm{d}\delta\ \rho_e(\theta'_x,\theta'_y,\delta) F_n(K;\ \theta_x - \theta'_x, \theta_y - \theta'_y)\ sinc^2(\pi n N(E/E_{res} - 1)) \quad \text{(S7)}$$

$$F_{cone}(K,n) = 1.431 \times 10^{14}\ N\ I[\mathrm{A}]\ F_n(K) \quad \text{(S8)}$$

Layer	Backend	When it fires	Latency	Physical model
0 (fast-path)	Closed-form JavaScript (sync)	Always, immediate	<1 ms	Closed-form fast-path estimate, Eq. (S8)
1 (GPU)	WebGPU WGSL compute shader	WebGPU available (tried first)	1-2 s	Slit-acceptance five-fold integral, Eq. (S7); FP32
2 (lookup)	Precomputed FP64 JSON table	No WebGPU; default WB slit, K in [0.20, 2.75]	<1 ms (interpolation)	Slit-acceptance five-fold integral, Eq. (S7); FP64 (offline)
3 (worker)	Web Worker (CPU thread)	No WebGPU and lookup miss	5–30 s	Slit-acceptance five-fold integral, Eq. (S7); FP64

Layers 1–3 implement the identical algorithm (an independent JavaScript implementation of the angle-dependent far-field amplitude of Section S3.2) and return the same slit-acceptance quantity (cross-validation figures at the end of this paragraph), so the WebGPU layer is a latency optimization rather than a correctness requirement: when WebGPU is unavailable the platform falls through to the lookup table or the CPU worker automatically, and the displayed flux is therefore reproducible across hardware. If the WebGPU device becomes ready only after the lookup or worker has already supplied an interim value, the engine re-runs the GPU pass and overwrites that value with the GPU-computed one. The fast path (Layer 0) returns the closed-form estimate of Eq. (S8), which combines the Kim (1989) central-cone flux constant 1.431×10^{14} with the flux-density coupling $F_n(K)$; relative to the full Kim central-cone expression it omits the $(1 + K^2/2)/n$ factor, and it includes neither the white-beam-slit acceptance nor the finite-beam suppression $S(K,n)$; it is explicitly tagged [fast] and is replaced by the converged white-beam-slit value within 1–2 s. The WebGPU kernel is split into eight sub-dispatches so each completes within the Windows GPU watchdog (TDR) limit; on device loss the engine reverts to the CPU layers. Cross-validation: the FP64 lookup table agrees with the SPECTRA solver to ≤1% (n = 3–23, K = 1.0/1.87/2.5), the FP32 WebGPU kernel agrees with the FP64 reference to a mean of 0.03% (f32 round-off only), and the Web Worker

reproduces the lookup to bit-exact level (validation/run_flux_acceptance_reference_grid.py and validation/phase3p6_3way_plot.png in the released repository).

S3.7 Closed-form fast path versus white-beam-slit-acceptance flux comparison

This subsection quantifies the difference between the two flux definitions that the engine can report for the same harmonic. The fast-path value is the closed-form estimate of Eq. (S8) (onAxisFlux), returned synchronously in under a millisecond; it is what the engine reported end to end before the four-layer cascade of Section S3.6 was introduced, and what the interface flashes briefly under the [fast] tag. The slit-acceptance value is the SPECTRA-validated angular amplitude integrated by a five-fold numerical quadrature over the white-beam-slit acceptance (halfH = halfV = 21.58 μrad), the electron-beam divergence and the energy spread; it is the converged quantity cited in manuscript Table 2 and Section S3.5. All three converged back-ends of the cascade return this same slit-acceptance value (Section S3.6).

The fast-path-to-slit-acceptance ratio quantifies the difference between these two quantities. Across the operating harmonics it ranges from 1.07 (5 keV, n = 3) to 10.6 (25 keV, n = 11; tabulated in Table S13). It is not a normalization discrepancy: it combines the physical difference between a filament central-cone estimate and the finite-beam, slit-integrated quantity reported by SPECTRA and by manuscript Table 2 with the $(1 + K^2/2)/n$ factor that Eq. (S8) omits.

The [fast] tag is displayed only as the sub-millisecond initial render so that the panel responds instantly; within roughly 100 ms to 2 s (or, on the CPU Web Worker fallback path, after its 5–30 s evaluation) the converged [gpu], [lookup] or [worker] value overwrites it, so the value a user records once the computation completes is the slit-acceptance value.

S3.8 Si(111) Darwin width

For the symmetric Si(111) Bragg case the angular Darwin width (Authier, 2001) is

$$\omega_D = 2\frac{|\chi_H|}{sin(2\theta_B)} \tag{S9}$$

where χ_H is the Fourier component of the crystal susceptibility for the (111) reflection and θ_B is the Bragg angle. The crystal susceptibility χ_H and Bragg angle in Eq. (S9), and the unit-cell volume entering the evaluation of the susceptibility, are extracted from the crystalpy library (Guigay & Sanchez del Rio, 2024), so that the analytical reference uses exactly the same crystal parameters as a crystalpy dynamical-diffraction calculation; the comparison with the engine over 5–25 keV is given in main-text Fig. 3(b) and Table S10.

S3.9 Displayed detector signal and absolute count rate

The engine's displayed detector signal is a relative quantity derived from the on-axis density, not a calibrated absolute count rate. For an ion chamber, conversion to an absolute count rate follows a standard chain: the power absorbed in the active gas, set by its tabulated attenuation at the photon energy, divided by the mean ion-pair energy of the fill gas gives the ion current, which the transimpedance amplifier gain converts to the recorded signal. This chain has established reference implementations, for example the ionization-chamber flux calculator of XAFSmass (Klementiev & Chernikov, 2016) and the web-based ion-chamber flux calculators operated at CHESS (https://www.chess.cornell.edu/users/technical-resources/calculators/ion-chamber-flux-calculator) and SSRL (https://www-ssrl.slac.stanford.edu/smbin/photonfluxcalc.pl). Introducing such a model into the engine and calibrating it against measured ion-chamber currents is identified as future work.

S3.10 Focused-spot construction

The focused spot at the sample is not taken as a single diffraction-limited value but is constructed as the convolution of three physical contributions: the geometrically demagnified source, the coherent Fraunhofer diffraction of the secondary source aperture (SSA), and the diffraction of the finite KB acceptance aperture,

$$I_{focus}(x,y) = (I_{src} \otimes P_{SSA} \otimes P_{KB})(x,y) \tag{S10}$$

where I_{src} is the source intensity, sampled from the SPECTRA-validated source model of Section S3.5 and demagnified by the object-to-image distance ratio of each focusing plane (element positions in Table S8); P_{SSA} is the Fraunhofer diffraction pattern of the SSA opening at the sample plane; and P_{KB} is the diffraction of the finite KB acceptance aperture. Neither diffraction kernel is evaluated in closed form: both are computed by the hybrid ray-wave procedure described below. The KB mirrors are treated as ideal elliptical reflectors, so mirror slope and figure errors are not included; the convolved spot is therefore an idealized, diffraction-limited estimate whose absolute value at the sample plane is qualified by the accuracy scope discussed in Section 3 of the main text.

In the Monte Carlo engine this construction is evaluated ray by ray rather than as an analytic convolution. Rays are sampled from the effective-Gaussian source size and divergence distributions of Section S3.5, the divergence including the energy-spread broadening, and each ray is propagated element by element: apertures clip rays (the white-beam slit additionally applies per-ray angular kicks sampled from the single-slit sinc-squared distribution), mirror and crystal reflections multiply the ray weight by the Fresnel and Darwin reflectivities (the latter from Section S3.8), and the KB mirrors apply the exact ellipsoidal reflection geometry.

Diffraction at the secondary source aperture and at the KB acceptance is applied as a hybrid ray-wave correction, using the same hybrid screen method as the SHADOW/OASYS toolchain (Shi et al., 2014), which combines geometric ray tracing with coherent wavefront propagation. For each aperture the procedure runs in five steps. (i) The footprint of the surviving rays at the aperture is binned into a one-dimensional histogram of up to 200 bins and interpolated onto the wavefront grid, giving the ray density $I(x)$. (ii) Its square root is taken as the coherent wavefront amplitude,

$$A(x) = \sqrt{I(x)} \tag{S11}$$

(iii) A quadratic thin-lens phase is applied,

$$A'(x) = A(x)\, exp[-i\pi \frac{x^2}{\lambda f_{ff}}] \tag{S12}$$

where the focal length is the effective far-field distance

$$f_{ff} = \frac{D^2}{2 \times 0.88\, n_{peaks}\lambda} \tag{S13}$$

where D is the footprint extent at the aperture, λ the X-ray wavelength, and $n_{peaks} = 20$; this distance is chosen so that the propagated diffraction pattern is resolved with about $2n_{peaks}$ fringes across the computation window.

(iv) The wavefront is propagated over f_{ff} by FFT-based Fresnel transfer-function propagation,

$$E(x) = \mathcal{F}^{-1}\{\mathcal{F}[A'](\nu)\, exp(-i\pi\lambda f_{ff}\, \nu^2)\} \tag{S14}$$

where $\mathcal{F}$ denotes the Fourier transform, implemented by FFT, and v the spatial frequency. (v) The propagated intensity, converted to angle $\theta = x/f_{ff}$, is sampled by inverse cumulative-distribution sampling to draw per-ray angular kicks,

$$p(\theta) \propto |E(f_{ff}\theta)|^2,\ \ \theta_r = C_p^{-1}(u_r) \tag{S15}$$

where C_p is the cumulative distribution of $p(\theta)$ and u_r a uniform random number. The kick is added to the geometric ray direction; for the KB, the geometric focusing kick is first subtracted so that only the diffraction contribution is resampled. At the sample plane the weighted rays are histogrammed (with a fine 300 nm field-of-view marginal histogram for the nanofocus), and the FWHM and second moments are extracted from the marginal distributions with sub-pixel interpolation.

S4. NLP Action Pipeline Implementation Details

The seven-layer NLP action pipeline (Fig. 5) processes each user request through deterministic pre- and post-processing stages surrounding a single LLM inference call. This section provides implementation details beyond the overview in Section 4.

S4.1 Layer 1: Pre-processing

Pre-processing operates before the LLM call and generates structured hints that are appended to the user message.

Element detection. The system recognizes 16 element symbols (Ti through Pb) via regex word-boundary matching in English and Korean alias lookup (e.g. “구리” → Cu, “철” → Fe). For each detected element, all absorption edges (K, L1, L2, L3) are looked up in an internal edge database and checked against the beamline’s 5–25 keV range. Out-of-range edges generate explicit warnings appended to the LLM input (e.g. “[HINT: Ti L-edge = 0.453 keV is BELOW beamline minimum 5 keV]”).

Intent classification. A keyword-based classifier assigns one or more intent tags from 12 categories: motor, scan_xrf, scan_xanes, scan_xrd, alignment, mask_atten, ptycho, scan_advanced, setup, nano_scanner, hw_status, and knowledge_query. Command intents route to the LLM pipeline; knowledge intents route to the RAG branch.

Language detection. Unicode script analysis detects the input language (Korean, Japanese, Chinese, Arabic, Thai, Hindi by script; Latin-based languages by keyword). The detected language overrides the browser’s navigator.language setting and is passed to the LLM for response generation.

S4.2 Dynamic Prompt Composition

Rather than using a fixed few-shot prompt, the system dynamically selects relevant examples based on detected intents. For each intent category, 1–3 representative input/output pairs are drawn from a curated example pool. This reduces prompt length (typically 40–60% of the full example set is sufficient) and improves accuracy by removing irrelevant examples that could confuse the LLM.

S4.3 Layer 3: Post-processing

Post-processing applies five deterministic corrections to the raw LLM output:

1. **Fuzzy function name matching.** Case-insensitive matching against 47 registered functions (e.g. “settargetenergy” → “setTargetEnergy”). Unmatched names are removed as hallucinations.
2. **Energy-set guard.** If the user intent is energy change only (not scan), scan actions (quickXanes, quickXafs) are replaced with setTargetEnergy.
3. **Automatic alignment insertion.** If setTargetEnergy changes energy by more than 1 keV from the current value, a runFullAlignment action is inserted after the energy change.

4. **Experiment function deduplication.** Virtual experiment functions (quickRaster, quickXanes, quickXafs) call startExperiment() internally, so redundant queueStart and setTargetEnergy actions are stripped.
5. **Missing queueStart addition.** If the last action is a scan function without a following queueStart, one is appended.

S4.4 Layer 4: Empty Response Retry

If the LLM returns an empty action list with a non-empty explanation (typically a clarification question), the system retries once with explicit guidance: “The user is requesting a beamline action. Generate at least one function call.” If the retry also fails, the original explanation is returned to the user.

S4.5 Layers 5–7: Validation Chain

Layer 5 (Energy range). Removes setTargetEnergy actions with energy values outside 5–25 keV and adds a user-visible warning.

Layer 6 (Domain rules). Validates function signatures (argument count and type), restricts homeMotor to KOHZU stages (BL10:SAM:CX/CY/CZ), and prevents incompatible motor-scan combinations.

Layer 7 (Sample preparation gate). In real hardware mode, blocks measurement actions (quickXanes, quickXafs, quickRaster) unless the user has confirmed sample mounting in the current conversation. This prevents empty-sample scans that could waste beam time.

S4.6 RAG Branch

Knowledge queries (detected by absence of command intents and presence of question markers) bypass the action pipeline. The RAG engine retrieves the top-5 relevant chunks from a pre-indexed documentation corpus (beamline specifications, motor ranges, technique descriptions) and generates a factual answer through the same LLM backend. RAG responses carry no actions and require no user confirmation.

S5. NLP Benchmark Details

The benchmark test suite comprises 228 prompts across 44 categories (per-category pass rates in Table S15), covering motor control, scans, alignment, multi-element experiments, edge cases, rejection of out-of-range requests, and virtual experiment workflows. Each test specifies a natural-language request (Korean, English, or Japanese), the expected function calls with parameter constraints, and a category label. A test passes if the generated actions match the expected sequence and all constraints are satisfied. Forty of the 44 categories achieved 100% pass rate.

S5.1 Expert-Identified Priority Improvement Areas

Independent expert review by two beamline scientists (Section 4 of the main text) produced a 30.9 percentage-point gap between automated pass rate (98.2%) and expert satisfaction (67.3%; Table S17). Cross-tabulating the two experts' per-response verdicts decomposes this gap: of the 228 responses, 115 (50.4%) were accepted by both reviewers, 36 (15.8%) were rejected by both (genuine deficiencies), and 77 (33.8%) were accepted by one reviewer but rejected by the other, giving an inter-expert agreement of 66.2% (Cohen’s kappa 0.23, fair). When each Not-OK judgment is attributed according to whether the other reviewer accepted the same response, 52% of the rejections reflect operator-preference ambiguity (an upper bound, since inter-expert disagreement also captures cases where one expert simply applies a stricter standard) and 48% reflect genuine deficiencies that both experts rejected; by failure type the ambiguity share is 49% for missing-parameter, 58% for wrong-action and 36% for unnecessary-action judgments. Detailed inspection of the Not-OK responses produced the following ten priority areas (approximate case counts in parentheses). Each item describes the observed gap, the number of affected test cases, and the mitigation being incorporated into the system prompt and post-processing pipeline.

P1 — Exposure time specification (~25 cases). Scan actions were generated without an exposure time argument. Mitigation: added a prompt rule instructing the agent to ask for or infer a default exposure time for every scan function call; post-processing layer injects a clarification dialog when the argument is missing.

P2 — Energy-dependent alignment criteria (~12 cases). Requests at new absorption edges did not always trigger full alignment. Mitigation: the existing energy-change guard ($dE > 1$ keV) was generalized to additionally trigger re-alignment when the target energy crosses a mirror-coating boundary or a DCM Bragg-angle threshold.

P3 — Scan parameter negotiation (~10 cases). Raster/line scan step size, range, and point count were filled with defaults without confirming with the user. Mitigation: a prompt rule pairs

step size to the current focused beam size (Nyquist criterion) and flags cases where the requested density is inconsistent with the physical beam.

P4 — Multi-element XRF energy selection (~8 cases). Energy was not always set above the highest absorption edge among the requested elements. Mitigation: post-processing rule validates energy >= max(edge_energies) + 1 keV for any multi-element XRF request.

P5 — Focusing optics and beam size (~6 cases). Nano-XRF requests sometimes proceeded without explicit focusing-optics selection. Mitigation: the system prompt now requires the agent to confirm KB/zoneplate/CRL selection and target beam size for any sub-micron request.

P6 — Setup vs execute disambiguation (~3 cases). Instructions such as "set it up for Fe XAFS" were interpreted by one expert as parameter staging only and by another as scan queuing. Mitigation: prompt disambiguation rule — "setup/prepare" language stages parameters and stops; "start/measure/run" language queues and starts the scan.

P7 — Mirror coating change (Pt L-edge, ~3 cases). The Pt L3-edge at 11.56 keV overlaps with Pt-stripe M2 absorption. Mitigation: prompt rule flags Pt L-edge measurements and recommends switching M2 stripe to Rh before the scan.

P8 — Simultaneous XRF/XRD measurement (~3 cases). Some responses advised sequential measurement where simultaneous is possible. Mitigation: system prompt updated with a statement that the ID10 endstation mounts XRF and XRD detectors at different scattering angles, allowing simultaneous collection.

P9 — Info versus action distinction (~5 cases). Questions that asked for values (e.g. "what energy should I use?") were answered by executing an action. Mitigation: Layer 1 intent classification now routes info-pattern inputs (question markers, "how much", "what is") to the RAG branch by default.

P10 — XRD technique details (~4 cases). XRD requests did not always include Bragg angle or accessible q-range information. Mitigation: prompt rule adds q-range calculation and a sensible default 2θ range based on target energy and sample-to-detector distance.

These ten areas summarize the most frequent patterns among the responses that at least one expert reviewer judged Not OK against the automated pass verdict; together they cover about one third of the 228 cases, and because a single case can fall under more than one area, the approximate per-area counts above overlap and sum to slightly more than the number of distinct cases covered. Each mitigation is implemented as an additive rule in either the system prompt or the Layer 3–7 post-processing pipeline described in Section S4, preserving the deterministic and stateless structure of the validation chain.

S6. Latency Measurement Results

S6.1 Scan event pipeline

Measurement: 51-point energy scan (9.9–10.1 keV), 57 documents in total (53 event documents). The scan event pipeline achieves a sub-millisecond median latency on localhost (Table S1; server and client co-located, so the values characterize software-serialization overhead and Python time.time() timer granularity rather than facility-network performance, and serve only as a software floor). The realistic remote measurement is the Remote path (a client reading from the server over a routed network with NAT; illustrated in Figure S2 and described in S1.3), reported in main-text Table 4: the PV write-to-client-receive round trip, which is single-clock measurable directly, has a median of 91 ms, bounded by the server-side periodic PV broadcast loop. One-way scan and WebSocket-transport latencies on the Remote path are not single-clock measurable; they are estimated with the clock-offset procedure of Section S1.3, giving a Remote-path one-way scan total of 2.3 ms (median), reported as an estimate rather than an operational transport value. The measurement script is released as tools/measure_latency.py and the per-scenario raw JSON outputs are included in the public repository.

S6.2 PV monitoring pipeline

Measurement: 23 PV update events over 10 seconds, motor oscillation on BL10:DCM:DTheta2.

The WebSocket transport latency is negligible (< 1 ms). The total motor-write-to-client latency is dominated by the server's PV broadcast-loop timing (Table S2): when a write coincides with a broadcast cycle the latency is near-zero, and when it falls between cycles it approaches the 100 ms loop interval. The measured localhost median is about 30 ms (a write lands, on average, partway through the cycle); the event-driven Channel Access bridge itself adds negligible latency.

S6.3 Per-event raw data

Timestamps are shown as seconds within the measurement epoch. The raw per-event timestamps are provided in Table S3; the complete datasets are available in latency_results.json.

S6.4 Measurement Script

The latency measurement script (tools/measure_latency.py) is included in the repository. To reproduce: (1) start the soft IOC and server with Bluesky enabled, (2) run the script with default parameters, (3) the script connects via WebSocket, submits a 51-point energy scan, records all timestamps, and outputs latency_results.json with per-event breakdown. The measurement client and server run on the same machine (localhost) to isolate transport overhead from

network latency. For the Remote scenario, the same script is run from a separate host and begins with the TCP round-trip probe of Section S1.3 to estimate the cross-host clock offset.

S7. System Architecture Details

S7.1 Three-layer data flow

The system consists of three layers communicating via standard protocols:

Layer 1: Browser Client (JavaScript, ES5)

WebSocket (RFC 6455, JSON)

| /ws/pv -- PV read/write/subscribe

| /ws/scan -- Bluesky plan submit/events

| /ws/chat -- NLP agent communication

Layer 2: Python Server (asyncio + websockets)

Channel Access (TCP, port 5064)

| caproto threading client

Layer 3: EPICS Soft IOC (caproto)

86 motor records (FakeMotor) 9 status PVs (ring current, BPM, ion chamber)

S7.2 Scan event pipeline

When a Bluesky scan plan is executed, scan events traverse the following path:

1. **RunEngine** executes the plan generator, moving ophyd EpicsMotor devices and reading detectors via Channel Access.
2. **Document callback** (BlueskyRunner._document_callback()) is invoked by the RunEngine for each document (start, descriptor, event, stop).
3. **Async scheduling**: the callback constructs a JSON-serializable event dict and schedules it on the asyncio event loop via asyncio.run_coroutine_threadsafe().
4. **WebSocket broadcast** (broadcast_scan_event()): the server serializes the event to JSON and sends it to all connected scan and chat clients.
5. **Client receipt**: the browser onmessage handler parses the JSON and dispatches to handleScanEvent() for chart updates and _enhancedScanEventHandler() for progress panel updates.

S7.3 PV monitoring pipeline

PV updates follow a periodic server-broadcast path:

1. **PV change detection**: in simulation mode, the PVStore detects value changes during its scan() cycle. In hybrid/real EPICS mode, the Channel Access bridge receives value changes through caproto subscribe callbacks (event-driven, no polling).
2. **Broadcast loop** (pv_broadcast_loop()): runs at 100 ms intervals, collects changed PVs, and sends per-client filtered updates via WebSocket.
3. **Client receipt**: the browser onmessage handler dispatches to handlePVUpdate() for motor panel and beamline layout updates.

S7.4 Zero-change hardware transition

The transition from virtual to real-hardware operation requires only: (1) stopping the caproto soft IOC, (2) updating the EPICS_CA_ADDR_LIST environment variable to point to the hardware IOC network. No changes to client code, ophyd device definitions, or scan plans are needed. This is possible because the soft IOC uses caproto FakeMotor with record='motor' (providing the complete EPICS motor record field set), the ophyd layer uses standard EpicsMotor without custom wrappers, and PV naming follows a consistent convention (BL10:<Device>:<Axis>).

S8. Scan Timing Analysis

S8.1 Energy scan (51 points, 9.9–10.1 keV)

The 51-point energy scan completed in approximately 7.8 seconds, yielding: - Per-point interval: ~150 ms (motor move + readout) - Cumulative event transport overhead: 53 events x 0.4 ms mean = ~21 ms - Transport overhead as fraction of total scan time: 21 ms / 7800 ms = 0.27%.

For a production scan with 1 s per-point integration time (about 58 s in total, of which ~51 s is integration), the transport overhead would be < 0.05%.

S9. Demonstration Movie (Movie S1)

Movie S1 (Movie_S1.mp4) is a continuous screen recording of the browser client that demonstrates the integrated workflow of Sections 2–5 of the main text in a single session. The recording proceeds in the following order, with the interactive SVG beamline layout and the view settings adjusted between steps to follow the optical path: (i) a knowledge query is typed into the chat panel in Korean, asking the agent to “이 빔라인에 대해 간단히 설명해(briefly describe the beamline)”, and is answered by the retrieval-augmented generation branch of the NLP pipeline (Section S4.6); (ii) the Kirkpatrick–Baez beam-defining slits are aligned; (iii) the sample-stage CX motor is moved by 1 mm through the standard motor interface; (iv) an X-ray absorption fine-structure (XAFS) scan of a TaN sample is requested and executed; (v) the secondary source aperture (SSA) is closed to 5 µm × 5 µm and the same TaN XAFS scan is repeated, showing how the changed secondary source propagates into the measured signal; (vi) a two-dimensional X-ray-fluorescence raster map of the Siemens-star resolution target is acquired; and (vii) the platform is switched from simulation to real hardware mode through the same interface.

Supplementary Tables

Table S1. Scan event latency statistics. **Measured on localhost; entries below the ~1 ms time.time() clock resolution are shown as <1 (a software floor, not a physical zero).**

Metric	Total (callback to client) (ms)	Callback to send (ms)	Send to WS send (ms)	WS send to client (ms)
Count	53	53	53	53
Min	<1	<1	<1	<1
Max	2.0	<1	2.0	1.0
Mean	0.4	<1	0.1	0.2
Median	<1	<1	<1	<1
Std	0.6	0.0	0.4	0.4
P10	<1	<1	<1	<1
P90	1.0	<1	1.0	1.0

Table S2. PV monitoring latency statistics (ms). **Measured on localhost; entries below the ~1 ms time.time() clock resolution are shown as <1 (a software floor, not a physical zero). Statistics are computed from unrounded timestamps.**

Metric	WebSocket transport (send to recv)	Total (motor write to recv)
Count	23	23
Min	<1	<1
Max	1.0	109.5
Mean	<1	28.7
Median	<1	31.4
Std	0.2	22.4
P10	<1	<1
P90	<1	32.0

Table S3. PV monitoring raw data (all 23 events).

#	Value	Write (s)	Scan (s)	Send (s)	Recv (s)	WS lat (ms)	Total lat (ms)
1	0.000	210.544	210.653	210.653	210.653	0.0	109.5
2	0.001	211.075	211.075	211.075	211.076	1.0	1.0
3	0.000	211.484	211.516	211.516	211.516	0.0	31.8
4	0.001	211.928	211.960	211.960	211.960	0.0	31.5
5	0.000	212.369	212.401	212.401	212.401	0.0	31.5
6	0.001	212.808	212.840	212.840	212.840	0.0	32.0
7	0.000	213.249	213.281	213.281	213.281	0.0	32.0
8	0.001	213.689	213.719	213.719	213.719	0.0	30.3
9	0.000	214.126	214.174	214.174	214.174	0.0	47.5
10	0.001	214.597	214.597	214.597	214.597	0.0	0.0
11	0.000	215.007	215.039	215.039	215.039	0.0	31.5
12	0.001	215.448	215.480	215.480	215.480	0.0	31.3
13	0.000	215.888	215.920	215.920	215.920	0.0	31.5
14	0.001	216.325	216.356	216.356	216.356	0.0	31.1
15	0.000	216.762	216.794	216.794	216.794	0.0	31.9
16	0.001	217.217	217.217	217.217	217.217	0.0	0.0
17	0.000	217.623	217.655	217.655	217.655	0.0	31.6
18	0.001	218.061	218.092	218.092	218.092	0.0	30.7
19	0.000	218.501	218.533	218.533	218.533	0.0	31.5
20	0.001	218.953	218.953	218.953	218.953	0.0	0.0
21	0.000	219.373	219.373	219.373	219.373	0.0	0.0
22	0.001	219.779	219.810	219.810	219.810	0.0	30.6
23	0.000	220.215	220.246	220.246	220.246	0.0	30.9

Table S4. Comparison of soft IOC simulation and real hardware IOC for each validated subsystem.

Property	Soft IOC (caproto)	KOHZU ARIES IOC	Sydor quadEM IOC	SmarAct TCP Bridge
IOC type	caproto FakeMotor (Python)	epics-base 7.0.8.1 + asyn + motor (C)	synApps quadEM (T4UDirect driver) (C++)	Custom TCP server (Python)
PV prefix	BL10:SAM:CX/CY/CZ	BL10:SAM:CX/CY/CZ	BL10:XBPM2:*	BL10:NANO:*
Protocol	Channel Access (port 5064)	Channel Access (port 5070)	Channel Access (port 5072)	TCP/JSON (port 5555)
Motor record	FakeMotor (velocity-limited simulated move)	Real motor record (asyn)	analog input (ai)	Custom position readback
Device connection	None (simulated)	TCP/IP to KOHZU controller (10.x.xx.xxx:y)	Ethernet to T4U electrometer (10.x.xx.xxx)	USB via notebook bridge (10.x.xx.xxx)
Axes/channels	3 motor (CX/CY/CZ)	3 motor (CX/CY/CZ)	4 current + 2 position	3 piezo + 3 interferometer
Resolution	Configurable	CX/CY: 0.5 um, CZ: 0.25 um	pA-level current	sub-nm (PicoScale)
Move settling	Instant	0.1-10 s (real motor)	N/A (readback only)	<100 ms
ophyd class	EpicsMotor	EpicsMotor (same)	EpicsSignalRO	Custom ophyd Device

Table S5. End-to-end verification results for each hardware subsystem.

Subsystem	PV Prefix	Command	Expected	Measured	Error
KOHZU CX	BL10:SAM:CX	move +2 mm	6.631 mm	6.631 mm	<1 MRES
KOHZU CY	BL10:SAM:CY	move +2 mm	7.338 mm	7.338 mm	<1 MRES
KOHZU CZ	BL10:SAM:CZ	move -2 mm	-7.820 mm	-7.820 mm	<1 MRES
Sydor BPM	BL10:XBPM2	read current	~4.5 pA	4.5 pA	nominal
SmarAct Ch0	BL10:NANO:SX	jog +100 nm	+100 nm	+100 nm	<0.1 nm

Table S6. Hardware validation coverage.

Subsystem	Test	Result	Notes
KOHZU CX	Jog +2 mm, -2 mm	Position error < 1 MRES (0.5 um)	Tested via browser UI motor control
KOHZU CY	Jog +2 mm, -2 mm	Position error < 1 MRES (0.5 um)	Same ophyd EpicsMotor as soft IOC
KOHZU CZ	Jog +2 mm, -2 mm	Position error < 1 MRES (0.25 um)	ZA07A-V1F01 stage
Sydor BPM	Read 4 electrode currents	~pA level (no beam, leakage current)	quadEM Direct IOC
SmarAct ch0	Closed-loop jog +100 nm	MCS2/PicoScale agreement < 0.1 nm	DDI feedback loop
Browser UI	Switch Virtual/Real mode	All controls operational, no code change	Zero-change strategy confirmed
Bluesky	beam_check plan on HW PVs	Completed without modification	Same ophyd devices as simulation

Table S7. KLS storage ring and ID10 source parameters (Section 3 of the main text).

Parameter	Value	Unit
Ring energy	4.0	GeV
Stored current	400	mA
Horiz. emittance	62	pm·rad
Vert. emittance	6.2	pm·rad
Coupling ratio	10	%
Energy spread	1.20×10^{-3}	dimensionless
Beta function (H)	6.334	m
Beta function (V)	2.841	m
Undulator period	24	mm
Number of periods	123	count
Total length	2.952	m
K range	0.01-2.5	dimensionless
Energy range	5-25	keV

Table S8. BL10 optical element positions and functions (Section 3 of the main text).

Element	Function	Distance (m)
IVU24	Source	0
Fixed mask	Power limiting	17.0
Movable mask	Aperture control	22.0
WB slit	White beam shaping	27.8
Attenuator	Flux control	28.3
M1 mirror	Focusing (V)	29.0
Double-crystal monochromator	Energy selection	30.4
M2 mirror	Focusing (H)	32.0
SSA	Secondary source	58.0
KB-V	Nano-focusing (V)	149.69
KB-H	Nano-focusing (H)	149.90
Sample	Experiment	150.0

Table S9. Monte Carlo convergence study at 10 keV (Section 3 of the main text).

Rays	$FWHM_H$ (nm)	$FWHM_V$ (nm)	$Centroid_H$ (μm)	Total flux (rel.)	Compute time (ms)
10,000	44.3	59.6	0.0024	0.996	25
50,000	45.7	60.5	0.0014	1.002	120
100,000	46.8	61.7	0.0009	1.003	350
500,000 (ref.)	47.3	63.0	0.0004	1.000	1,850

Table S10. Si(111) Darwin width: computed (this work, Zachariasen–Authier formulation) vs the Authier (2001) analytical reference of Eq. (S9) (crystal parameters from the crystalpy library, Guigay & Sanchez del Rio, 2024), over the 5–25 keV operational range (Section 3 of the main text). Deviations are computed from unrounded widths.

Energy (keV)	Authier (2001) (μrad)	This work (μrad)	Dev (%)
5	58.7	55.8	-4.9
8	34.3	33.1	-3.6
10	27.0	26.2	-3.0
12	22.2	21.7	-2.6
15	17.6	17.2	-2.2
20	13.1	12.9	-1.8
25	10.5	10.3	-1.6

Table S11. Undulator source size compared with SPECTRA at the representative operating point K = 1.87, over the odd harmonics n = 3–15 (operational band n = 3–11). The tabulated values are the effective-Gaussian RMS source sizes Σ_x (horizontal) and Σ_y (vertical) of Eq. (S5): the natural single-electron size in quadrature with the electron-beam size. The comparison is independent of the flux validation and does not use the precomputed lookup table.

Harmonic	Energy (keV)	Σ_x, this (μm)	Σ_x, SPECTRA (μm)	Dev.	Σ_y, this (μm)	Σ_y, SPECTRA (μm)	Dev.
3	6.91	19.99	19.98	+0.01%	4.93	4.92	+0.22%
5	11.52	19.92	19.92	+0.01%	4.65	4.64	+0.15%
7	16.12	19.89	19.89	+0.01%	4.53	4.52	+0.11%
9	20.73	19.87	19.87	+0.00%	4.46	4.45	+0.09%
11	25.34	19.86	19.86	+0.00%	4.41	4.41	+0.08%
13	29.95	19.86	19.86	+0.00%	4.38	4.37	+0.07%
15	34.55	19.85	19.85	+0.00%	4.35	4.35	+0.06%

Table S12. Undulator angular divergence compared with SPECTRA at K = 1.87 over n = 3–15, from the same independent physics calculation. Σ'_x and Σ'_y are the RMS divergences of Eq. (S6), the natural divergence including the energy-spread broadening of Section S3.5.

Harmonic	Energy (keV)	Σ'_x, this (μrad)	Σ'_x, SPECTRA (μrad)	Deviation	Σ'_y, this (μrad)	Σ'_y, SPECTRA (μrad)	Deviation
3	6.91	9.42	9.43	−0.12%	9.01	9.02	−0.13%
5	11.52	9.15	9.16	−0.07%	8.73	8.74	−0.08%
7	16.12	9.05	9.05	−0.05%	8.62	8.62	−0.05%
9	20.73	8.99	9.00	−0.04%	8.56	8.56	−0.04%
11	25.34	8.96	8.96	−0.03%	8.52	8.52	−0.03%
13	29.94	8.93	8.93	−0.02%	8.50	8.50	−0.03%
15	34.55	8.92	8.92	−0.02%	8.48	8.48	−0.02%

Table S13. Closed-form fast-path estimate (Eq. (S8)) versus white-beam-slit-acceptance (integral) flux for the IVU24 source (E = 4.0 GeV, I = 0.4 A, λ_u = 24 mm, N = 123), over photon energies 5–25 keV and harmonics n = 3–11. Slit-acceptance values are the peak of the slit-transmitted harmonic spectrum (slightly red-shifted from the on-axis resonance; Section S3.3), not the partial flux at the fixed nominal energy. Flux in photons s^{-1} (0.1% bandwidth)$^{-1}$.

E **(keV)**	***n***	***K***	**Fast-path (CPU)**	**Slit-acceptance (GPU)**	**Ratio (fast-path / slit-acceptance)**
5	3	2.366	2.75e+15	2.58e+15	1.07
5	5	3.266	2.47e+15	OOR	—
5	7	3.966	2.26e+15	OOR	—
5	9	4.560	2.11e+15	OOR	—
5	11	5.085	1.99e+15	OOR	—
8	3	1.658	2.89e+15	1.82e+15	1.59
8	5	2.432	3.04e+15	1.50e+15	2.03
8	7	3.013	2.96e+15	OOR	—
8	9	3.500	2.85e+15	OOR	—
8	11	3.926	2.76e+15	OOR	—
10	3	1.341	2.35e+15	1.20e+15	1.97
10	5	2.081	2.98e+15	1.21e+15	2.46
10	7	2.620	3.08e+15	9.67e+14	3.18
10	9	3.066	3.07e+15	OOR	—
10	11	3.454	3.02e+15	OOR	—
15	3	0.730	4.33e+14	1.50e+14	2.89
15	5	1.490	1.83e+15	5.10e+14	3.58
15	7	1.977	2.45e+15	5.34e+14	4.58
15	9	2.366	2.76e+15	4.81e+14	5.74
15	11	2.699	2.93e+15	4.31e+14	6.78
20	3	n/a	n/a	n/a	—
20	5	1.080	5.68e+14	1.22e+14	4.66
20	7	1.560	1.30e+15	2.18e+14	5.95
20	9	1.923	1.80e+15	2.41e+14	7.46
20	11	2.228	2.15e+15	2.48e+14	8.67
25	3	n/a	n/a	n/a	—
25	5	0.730	5.55e+13	9.77e+12	5.68
25	7	1.243	4.47e+14	6.17e+13	7.24
25	9	1.600	8.79e+14	9.65e+13	9.11
25	11	1.890	1.25e+15	1.18e+14	10.61

OOR (out of range) marks harmonics whose deflection parameter K exceeds the offline-precomputed lookup grid (K_{max} = 2.75); the GPU layer still evaluates the correct converged value on demand at run time, but the offline tabulation was not extended this far, so the slit-acceptance cell is left blank; these K values also exceed the IVU24 operating range itself

(Table S7), so they are unreachable operating points listed only to complete the fast-path map. n/a marks (E, n) combinations for which no real K solves $E = n \cdot E_1(K)$ for the IVU24 source.

Table S14. Prompt engineering vs model scaling effectiveness. The three rows are independent improvement axes (prompt revision within a fixed engine; model scaling within a fixed engine; engine substitution at a fixed model size) and are not strictly chainable because the Ollama and vLLM engines give slightly different baselines for the same Qwen3 model. Each percentage is reported on the benchmark suite as it existed at that development stage (v1.0: 82 tests; v2.0: 154; v2.1: 178; final: 228), so the deltas in rows 1 and 3 compare across suites; the underlying counts are given in each cell. The 98.2% headline reported in the Abstract and Table 3 is the final 32B Qwen3 model on vLLM with the v2.1 prompt and the full deterministic post-processing pipeline applied (the same configuration used for all 228-case results), recovering ~0.4 percentage points over the v2.1 vLLM intermediate (97.8% in row 3, measured on the then-current 178-test suite). The 92.9% baseline in row 3 is a 32B vLLM run that coincidentally equals the 235B model in Table 3 (both 143/154); these are different models and runs.

Change	Accuracy change	Cost
v1.0 → v2.0 prompt engineering (8B)	+33.5 pp (56.1%, 46/82 → 89.6%, 138/154)	Zero compute cost
8B → 32B model scaling (Ollama, v2.0)	+8.5 pp (89.6%, 138/154 → 98.1%, 151/154)	+14 GB VRAM, +31 s latency
v2.0 → v2.1 prompt engineering (32B vLLM)	+4.9 pp (92.9%, 143/154 → 97.8%, 174/178)	Zero compute cost

Table S15. Per-category pass rates (Qwen3-32B vLLM, 228 tests).

Category	Tests	Pass	Category	Tests	Pass
Basic Motor Control	3	100%	Robustness	12	100%
Scans & Measurements	9	100%	Rejection	10	100%
Auto-alignment	4	100%	Korean Variants	9	100%
Multi-step Commands	1	100%	Signal Estimation	5	100%
Attenuator & Mask	4	100%	Beamline Knowledge	5	100%
Information Queries	3	100%	Experiment Planning	10	100%
Parameter Confirmation	2	100%	Experiment Planning (Adv.)	10	100%
Sample Scientist	2	100%	Real User Scenarios	12	100%
Catalyst Research	3	100%	Complex Multi-step	9	100%
Semiconductor	2	100%	SSA Control	3	100%
Geology	3	100%	Sequential	3	100%
Environment	2	100%	Question + Action	2	100%
Biology	2	100%	Partial Range	2	100%
Edge Cases	7	100%	Colloquial	4	100%
Beamline Operations	3	100%	Safety	2	100%
Materials Science	3	100%	Implicit Technique	3	100%
Held-out	15	100%	Analysis Intent	3	100%
Optimization	7	85.7%	Heavy Element	2	100%
Battery Research	5	80.0%	Experiment Preset	8	87.5%
Technique Selection	8	100%	Experiment Edge Cases	5	100%
Timing Feasibility	5	100%	Multi-technique Workflow	8	100%
Ptychography	6	100%	Workflow	2	50.0%

Table S16. Ollama vs vLLM inference comparison (Qwen3-32B).

Metric	Ollama (local)	vLLM (local, 2xA6000)
Model	Qwen3-32B	Qwen3-32B
Tests	154 (v2.0 prompt)	228 (v2.1 prompt)
Pass rate	98.1%	98.2%
Avg latency	67.0 s	21.6 s
Prefix caching	No	Yes
GPU memory	~20 GB (Q4)	~40 GB (FP16)

Table S17. Expert review summary and gap decomposition (two beamline scientists, 228 responses each).

Metric	Reviewer A	Reviewer B	Average
OK count	154	153	153.5
Not OK count	74	75	74.5
OK rate	67.5%	67.1%	67.3%
Inter-reviewer agreement	66.2% (151/228)		
Cohen's kappa	0.23 (fair)		
Both OK	—	—	115 (50.4%)
Both Not OK (genuine deficiency)	—	—	36 (15.8%)
Disagreement (operator-preference ambiguity)	—	—	77 (33.8%)
Rejections attributable to ambiguity	—	—	52% (48% genuine)

Supplementary Figures

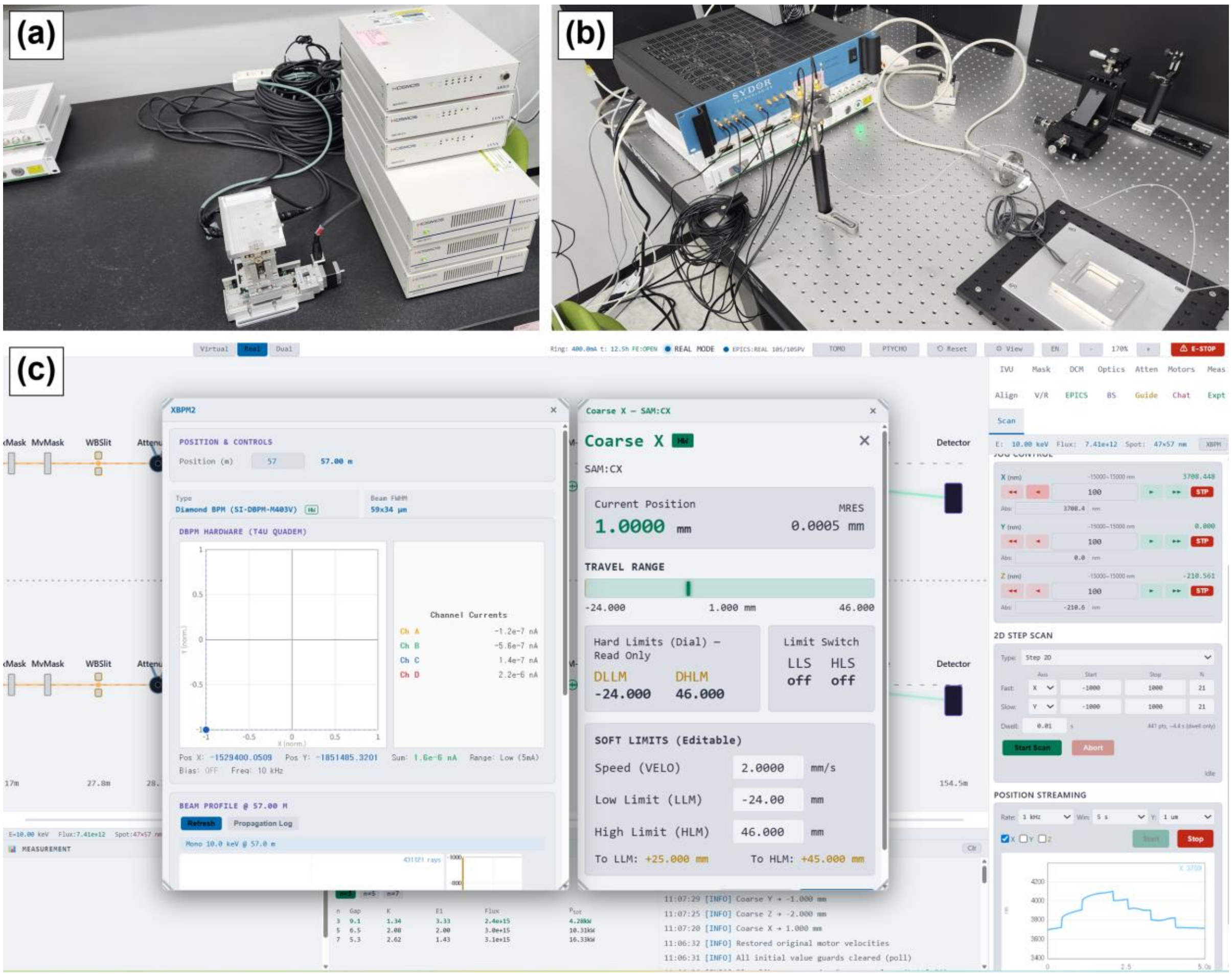

Figure S1. End-to-end validation of the zero-change hardware transition strategy. (a) KOHZU three-axis sample stage and its ARIES motor-controller stack. (b) Sydor diamond beam position monitor electronics and detector head, together with the SmarAct nano-positioning scan stage and PicoScale interferometer, on the optical test bench. (c) HANBIT browser client operating these subsystems in hybrid mode: real hardware subsystems run alongside the virtual soft IOC on the same UI, the only change from simulated operation being the EPICS CA address list. Two discrete hardware panels are shown: (left) the Sydor BPM via a synApps quadEM IOC, showing live per-channel currents (no-beam noise-floor readings at capture time) and the beam position; (right) the KOHZU stage as a standard EPICS motor record (.VAL/.RBV/.DMOV/.HLM/.LLM/.EGU/.VELO). The SmarAct MCS2 nano-positioner with PicoScale interferometer is represented on the right sidebar as nm-scale JOG CONTROL inputs and a live POSITION STREAMING readback, providing closed-loop position feedback over the TCP bridge through the same browser client.

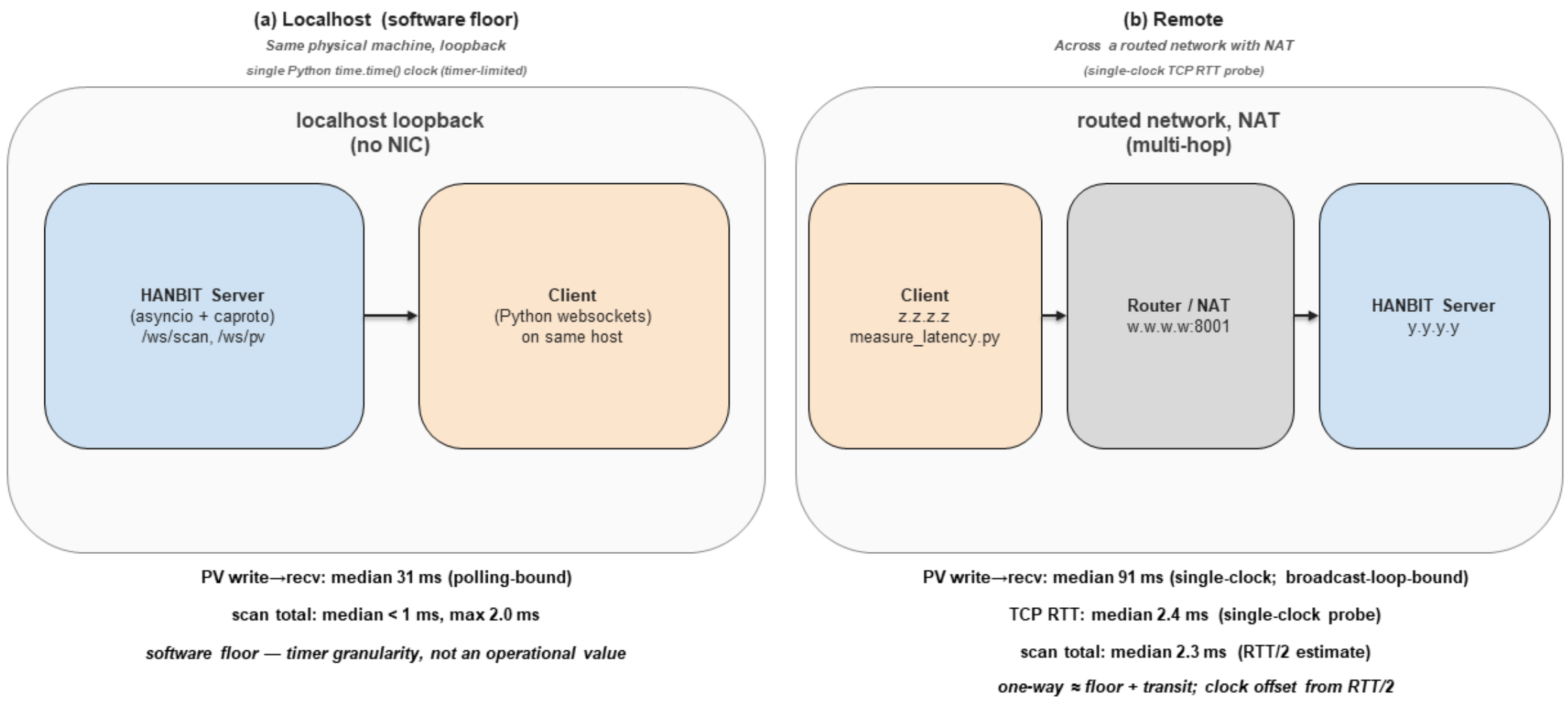

Figure S2. Network paths used for the two latency measurement scenarios. (a) Localhost (software floor): server and client on the same machine, communicating via loopback; the sub-millisecond medians are bounded by timer granularity and characterize software-serialization overhead, not network performance (Tables S1 to S3). (b) Remote: a client reading from the server over a routed network with NAT. The single-clock-measurable end-to-end metric is the PV write-to-client round trip (median 91 ms, bounded by the server-side periodic PV broadcast loop). One-way scan and WebSocket-transport latencies are not single-clock measurable; they are estimated by adding a cross-host clock offset from a single-clock TCP round-trip probe (median 2.4 ms over 30 HTTP GET requests, implying about 1.2 ms one-way transit), giving a one-way scan total of 2.3 ms (median), and reflect the serialization floor plus the network transit (not the transit alone). The measurement script is released as tools/measure_latency.py.

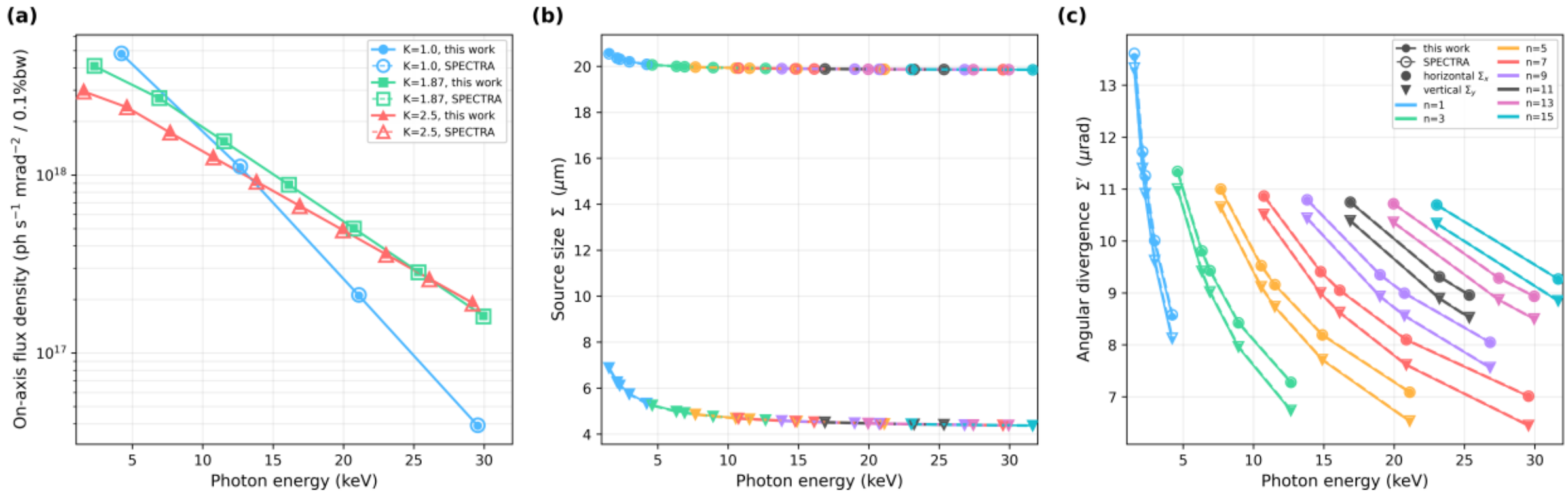

Figure S3. Undulator source model validated against SPECTRA for the KLS IVU24 (4.0 GeV, 400 mA, natural emittance 68.2 pm rad at 0.1 coupling, energy spread 1.20×10^{-3}; λ_u = 24 mm, 123 periods (2.952 m magnetic length)). (a) On-axis flux-density tuning curves at K = 1.0, 1.87 and 2.5: the engine (filled markers, solid) reproduces the SPECTRA peak flux density (open markers, dashed) of every plotted harmonic to within 0.5% mean (1.1% maximum) absolute deviation; the per-harmonic flux-density comparison at the K = 1.87 operating point is tabulated in main-text Table 2. (b) Source size Σ_x, Σ_y and (c) angular divergence Σ'_x, Σ'_y versus photon energy for odd harmonics n = 1–15 at K = 1.0, 1.5, 1.87, 2.0 and 2.5 (the SPECTRA-validated band; Tables S11 and S12 tabulate n = 3–15 at K = 1.87); mean absolute deviations are 0.09% (size) and 0.13% (divergence) over n = 1–15, K = 1.0–2.5. Circles denote the horizontal axis, triangles the vertical; colors denote the harmonic number. The vertical offset between adjacent-harmonic curves at a given photon energy is the undulator harmonic-transition structure of the source, which the engine reproduces.
